# EQ-5D vision bolt-on in macular degeneration: associations with visual measures and effect on utility differences and cost-effectiveness of stereotactic radiotherapy

**DOI:** 10.1186/s12955-025-02457-w

**Published:** 2026-07-14

**Authors:** Xuemin Zhu, Sarah Wordsworth, Chan Ning Lee, Hatem A. Wafa, Yanzhong Wang, Riti Desai, Lisa Ramazzotto, Barnaby C. Reeves, Timothy L. Jackson, Helen Dakin

**Affiliations:** 1 Nuffield Department of Population Health, University of Oxford, Old Road Campus, Headington, Oxford, OX3 7LF UK; 2 Oxford NIHR Biomedical Research Centre, University of Oxford, Oxford, UK; 3 Faculty of Life Sciences and Medicine, King’s College London, London, UK; 4 King’s Ophthalmology Research Unit, King’s College Hospital, London, UK; 5 Department of Population Health Sciences, School of Life Course and Population Sciences, King’s College London, London, UK; 6 Population Health Sciences, Bristol Medical School, University of Bristol, Bristol, UK

## Abstract

**Background:**

The UK STAR trial [Stereotactic Radiotherapy (SRT) for neovascular age-related macular degeneration (nAMD)] compared 16-Gray SRT (*n* = 274) with double-masked sham SRT (*n* = 137) in participants with chronic active nAMD. SRT reduced intravitreal anti-vascular endothelial growth factor (VEGF) re-treatments over two years of pro re nata ranibizumab, followed by two years of routine care. However, this resulted in worse best-corrected visual acuity (BCVA) in Years 3 and 4. This paper describes: associations between the EQ-5D vision bolt-on and visual acuity and patient-reported outcome measures; the impact of SRT on utilities with and without vision bolt-on; how the vision bolt-on affects the cost-effectiveness of SRT plus anti-VEGF versus anti-VEGF alone.

**Methods:**

Using data from the entire STAR trial cohort, we compared mean BCVA, EQ-5D-5L, and Visual Function Questionnaire-25 (VFQ-25) scores across the three levels of the EQ-5D vision bolt-on. We examined the relationship between BCVA and EQ-5D (with/without the bolt-on) and VFQ-25. An economic evaluation estimated the cost-effectiveness of SRT from a UK national health service perspective over two years and over four years. This used prospective data on EQ-5D-5L and eye-related direct healthcare use.

**Results:**

Participants reporting vision problems on the bolt-on had significantly worse BCVA, EQ-5D-5L, and VFQ-25 scores than those who did not. EQ-5D utilities (with and without the bolt-on) increased with BCVA but showed weaker correlations than VFQ-25 composite scores. Quality-adjusted life years (QALYs) did not differ significantly between treatment groups, with or without the bolt-on. The economic evaluation suggested SRT would reduce healthcare costs by £404 (95% CI: -£1282 to £2092) per patient at a four-year time horizon. The probability of SRT plus anti-VEGF being cost-effective at a £20,000 per QALY threshold was 65% at a four-year time horizon. Sensitivity analyses confirmed the robustness of incorporating the vision bolt-on did not alter the cost-effectiveness conclusion.

**Conclusions:**

Resource use data from the trial and routine follow-up could be used for future economic models. The vision bolt-on captured quality of life differences between participants but may not be sufficiently responsive to visual acuity decline in trials where only one eye is treated.

**Trial registration:**

ISRCTN12884465, registration date 01/12/2014.

**Supplementary Information:**

The online version contains supplementary material available at 10.1186/s12955-025-02457-w.

## Introduction

Age-related macular degeneration (AMD) is the most common cause of blindness in high-income countries [1, 2], affecting an estimated 67 million people in Europe and 196 million people worldwide [3, 4]. Neovascular AMD (nAMD) is responsible for the majority of severe vision loss associated with AMD [5]. The management of nAMD typically involves injecting anti-vascular endothelial growth factor (VEGF) agents to improve or stabilise vision. However, whilst effective, intravitreal injections are burdensome for patients and costly for health services.

By selectively targeting dividing and fast-growing cells, radiotherapy has the potential to inhibit the growth of new blood vessels in nAMD and therefore reduce the number of anti-VEGF injections required to control the disease. Accordingly, a commercial device (IRay, Carl Zeiss, Jena, Germany) was developed to precisely deliver highly collimated beams of stereotactic radiotherapy (SRT) as a one-off treatment. Two trials have evaluated the use of the IRay SRT device: IRay in conjunction with anti-VEGF treatment for patients with nAMD (INTREPID) [6] and stereotactic radiotherapy for neovascular age-related macular degeneration (STAR) [7]. These are the largest and longest-running trials of SRT for nAMD to date.

The trial findings present a complex picture. SRT reduced the number of ranibizumab retreatments at one year [6] and two years [7] without compromising best-corrected visual acuity (BCVA). However, the four-year follow-up showed worse BCVA in the SRT group compared to the sham SRT group (*p* < 0.01; eight-letter deficit) [8]. Visual Function Questionnaire-25 (VFQ-25) and EQ-5D-5L responses remained comparable between groups [8]. This disconnect between clinical and patient-reported measures raises questions about how the impact of vision impairment is measured.

EQ-5D-5L is the generic utility instrument most widely used in economic evaluations to inform healthcare decisions [9]. Its sensitivity is therefore critical, yet concerns have been raised about its ability to capture meaningful changes in some conditions, including vision impairments [10]. A three-level vision bolt-on item was developed in the mid-2010s to address this gap [11] and has been shown to better capture variation in visual impairment [12], although its sensitivity in interventional studies remains untested.

Simple costing analyses on STAR treatment costs have been reported previously [7, 8]. By Year 4, the SRT group in the STAR trial reported numerically more ocular adverse events, including cataracts, macular haemorrhage, and posterior capsular opacification. It is therefore essential to consider all healthcare resource use related to the eye, including the costs of managing adverse effects that were excluded from the previous costing analyses [7, 8].

Based on the STAR trial, this study aims to analyse: (a) the associations between the EQ-5D vision bolt-on and visual acuity measures and patient-reported outcome measures; (b) the impact of study intervention on utilities with and without a three-level vision bolt-on; (c) how the vision bolt-on affects the cost-effectiveness of SRT plus anti-VEGF versus anti-VEGF alone.

## Methods

### Data

The STAR study (NCT02243878; ISRCTN12884465) was a sham-controlled, double-masked, randomised UK trial evaluating the safety and efficacy of SRT in participants with pre-existing nAMD [7, 8, 13]. The trial was conducted in accordance with the Declaration of Helsinki, and all participants provided written informed consent. Participants were randomised 2:1 to receive either one-off 16-Gray SRT (*n* = 274) or sham SRT (*n* = 137) that was identical to active SRT but with 0 Gy dose. Both treatments were administered at one of three national treatment centres, followed by an intravitreal injection of 0.5 mg ranibizumab in both groups. Thereafter, participants were reviewed by local study teams every four weeks for two years with *pro re nata* (PRN) ranibizumab re-treatment, using pre-defined re-treatment criteria [13, 14]. After Year 2, participants remained masked but returned to routine care, following local pathways for monitoring and anti-VEGF drug selection and dosing, with full study evaluations conducted at the end of years three and four. This extended duration of observation aimed to assess long-term efficacy and safety (as radiation damage may occur later), and to obtain real-world data that may be more generalisable than on-trial results.

### Health-related quality of life (HRQoL) measures

STAR collected data on two patient-reported outcomes:

- The validated, preference-based EQ-5D-5L questionnaire comprises five questions covering mobility, self-care, usual activities, pain/discomfort, and anxiety/depression, as well as a visual analogue scale (VAS), on which respondents rate their overall health from 0 (worst imaginable) to 100 (best imaginable) [15]. Health status in these five domains was converted to an EQ-5D-3L utility using the Hernandez Alva crosswalk [16], reflecting the preferences of the UK general population [17]. A three-level vision ‘bolt-on’ question was also included, in which participants rated their vision (corrected by spectacles or contact lenses if needed) as having no problems, some problems, or extreme problems [11]. Based on a valuation study, we adjusted participants’ EQ-5D utility score based on the vision bolt-on responses by subtracting 0.0378 for some vision problems and 0.130 for extreme vision problems [18]. In a sensitivity analysis utility scores were estimated using the EQ-5D-5L value set for England [19], to further examine the sensitivity of utility to the vision bolt-on across value sets.
- The National Eye Institute VFQ-25 evaluates visual function across 12 domains, including general vision, near and distance activities, social functioning, mental health, role difficulties, and dependency. Each domain is scored from 0 to 100, with higher scores indicating better self-reported visual function. A composite score is calculated as the average of the vision-related domains (excluding general health), providing an overall summary of vision-related health status [20].

### Associations between quality of life measures and BCVA

To explore how quality of life measures relate to clinical visual function, we pooled responses from both trial groups at all timepoints and compared them with BCVA, the most widely used measure of visual function in AMD and in ophthalmic research [21]. BCVA was assessed using the Early Treatment Diabetic Retinopathy Study (ETDRS) letter chart, reflecting the number of letters correctly identified, with higher scores indicating better vision [22]. EQ-5D utility scores (with and without the vision bolt-on) and VFQ composite scores were plotted against BCVA using locally weighted scatterplot smoothing (LOWESS) to visualise how quality of life varies with BCVA in the better-seeing eye and worse-seeing eye [23]. EQ-5D dimension levels were also compared against BCVA using a box-and-whisker plot. To explore how vision and patient-reported outcomes vary across levels of self-reported vision using the EQ-5D vision bolt-on, we compared mean scores for each outcome measure (BCVA, EQ-5D, and VFQ-25) across the three levels of the vision bolt-on item. Differences between these levels were estimated using mixed effects linear regression for continuous outcomes (BCVA, EQ-5D utility, EQ-5D VAS, and VFQ-25) and multinomial logistic regression for ordinal EQ-5D responses. All models adjusted for sex at birth, baseline age, and the number of on-trial days affected by the COVID-19 pandemic, with a random intercept specified for each participant to account for within-subject correlation.

### Between-group differences in health-related quality of life

Between-group differences were compared using ordinary least squares linear regression and adjusted for sex at birth, age, EQ-5D utility, study eye BCVA and days affected by COVID-19 [31]. Cohen’s d effect sizes were calculated to quantify the magnitude of treatment-related differences in health outcome measures [24].

### Economic evaluation

The cost-utility analysis of SRT was the pre-specified aim of the STAR trial. We also examine how cost-effectiveness was affected by the use of the EQ-5D vision bolt-on from the United Kingdom (UK) National Health Service (NHS) perspective and evaluate how cost-effectiveness was affected by the use of the EQ-5D vision bolt-on. As pre-specified in the STAR trial protocol [13], the base-case economic evaluation compared the cost-effectiveness of SRT plus anti-VEGF with anti-VEGF alone at over two years (96 weeks), aligning with the trial’s primary endpoint. A secondary analysis extended the time horizon to four years (192 weeks). We included all participants as-randomised following the intention-to-treat principle, with missing data imputed using multiple imputation (detailed methods in appendix pp 8–27).

We used EQ-5D-5L utilities to calculate quality-adjusted life years (QALYs) for each participant as the area under the annual health utility curve over the study period. QALYs capture both the quality and quantity of life lived, and facilitate comparisons across different disease areas and populations within a healthcare system [25]. Utilities with the bolt-on were used in a sensitivity analysis.

The economic evaluation included all direct NHS resources related to the study eye. These included SRT, monitoring consultations, anti-VEGF administrations (covering both drug and administration), hospital care (covering outpatient consultations, outpatient procedures, and hospitalisations), primary care and concomitant medications (covering consultations and procedures in community settings, and concomitant medications). As per the pre-specified health economic analysis plan that we developed before unmasking (appendix pp 3–7), we included concomitant medications in the Medical Dictionary for Regulatory Activities (MedDRA) organ categories that (a) were considered a priori to be plausibly related to the study intervention and (b) showed a statistically significant difference in the number of new prescriptions in the first two years at the 0.1 level. In line with NICE guidance to exclude costs unrelated to the condition or technology of interest [17], admissions and consultations unrelated to the study eye were excluded from the costing analysis. Relatedness was adjudicated by a clinician with treatment allocation masked, and all excluded items are listed in the Appendix (pp 18–25). Participants reported resource use related to the study eye during each study visit. Unit costs were based on NHS data [26–30] and previous studies [7] (Appendix Table S1). The reference year for costs was 2021/22.

We estimated costs and QALYs separately for Years 1–2 (trial monitoring/retreatment protocol) and Years 3–4 (routine care). Costs and QALYs beyond Year 1 were discounted at an annual rate of 3.5%, following the National Institute for Health and Care Excellence (NICE) reference case [17]. We used non-parametric bootstrapping to quantify the uncertainty around costs and QALYs. Data from each imputed dataset were bootstrapped separately. Linear regression was used to adjust for potential baseline imbalance in sex at birth, age, EQ-5D utility, study eye BCVA and days affected by COVID-19 [31]. We assumed that the NHS is willing or able to pay up to £20 000 per QALY gained when interpreting cost-effectiveness ratios and estimating the probability that SRT plus anti-VEGF is cost-effective [17]. We conducted 24 stochastic sensitivity analyses to assess the robustness of the results, for example, varying the price of anti-VEGF drugs, SRT license fees, monitoring visit frequency and discount rates (appendix p 16).

All analyses were conducted in Stata version 17.0 (StataCorp, College Station, TX). With the exception of the test on concomitant medication categories, all analyses were interpreted using a 0.05 significance level.

## Results

### Associations between the EQ-5D, vision bolt-on, and VFQ and BCVA

At Year 4, 67% (218/326) of trial participants reported vision-related problems on the EQ-5D vision bolt-on (Table S3). Compared with those reporting “no problems” with vision on the bolt-on, participants reporting “some” or “extreme problems” showed significantly worse BCVA, EQ-5D utility and VAS scores, and VFQ-25 subscale and composite scores. For instance, mean EQ-5D utility valued using the Hernandez-Alva crosswalk was higher for people with better vision: 0.90 (no problems), 0.78 (some problems), and 0.60 (extreme problems). Utilities valued with the England 5 L set showed a similar pattern (Table S6).

Vision problems reported on the EQ-5D bolt-on were linked to greater limitations in all five EQ-5D domains (Table 1; *p* < 0.01). For example, those reporting “no vision problems” had an 86% (594/693) rate of no difficulty on usual activities, compared to 53% (502/950) for “some problems” and 21% (34/159) for “extreme problems” (Table S7).

**Table 1:** Coefficients of vision bolt-on on vision-related quality of life

	Vision (using glasses or contact lenses if needed)							
	Some problems				Extreme problems			
	Coefficient	SE	Z	*P* value	Coefficient	SE	Z	*P* value
BCVA: Better-seeing Eye	-2.62	0.46	-5.73	*p* < 0.01	-11.32	0.84	-13.41	*p* < 0.01
BCVA: Worse-seeing Eye	-5.23	0.81	-6.46	*p* < 0.01	-13.24	1.51	-8.79	*p* < 0.01
Equation 5D: Utility valued from Hernandez Alva crosswalk	-0.07	0.01	-7.83	*p* < 0.01	-0.20	0.02	-12.83	*p* < 0.01
Equation 5D: Utility using England 5 L value set	-0.05	0.01	-7.19	*p* < 0.01	-0.17	0.01	-13.05	*p* < 0.01
Equation 5D: Visual Analogue Scale	-4.17	0.71	-5.89	*p* < 0.01	-14.18	1.31	-10.84	*p* < 0.01
VFQ: General Vision	-13.87	0.84	-16.57	*p* < 0.01	-37.82	1.53	-24.79	*p* < 0.01
VFQ: Ocular Pain	-4.20	0.74	-5.67	*p* < 0.01	-11.03	1.36	-8.11	*p* < 0.01
VFQ: Near Vision	-12.15	0.85	-14.21	*p* < 0.01	-30.44	1.58	-19.25	*p* < 0.01
VFQ: Distance Vision	-8.18	0.86	-9.48	*p* < 0.01	-28.27	1.61	-17.60	*p* < 0.01
VFQ: Social Functioning	-5.22	0.80	-6.51	*p* < 0.01	-21.97	1.48	-14.83	*p* < 0.01
VFQ: Mental Health	-10.29	0.91	-11.31	*p* < 0.01	-31.31	1.68	-18.61	*p* < 0.01
VFQ: Role Difficulties	-11.41	1.07	-10.67	*p* < 0.01	-32.52	1.98	-16.41	*p* < 0.01
VFQ: Dependency	-6.95	0.96	-7.20	*p* < 0.01	-28.66	1.78	-16.07	*p* < 0.01
VFQ: Driving	-8.44	1.56	-5.42	*p* < 0.01	-30.01	3.23	-9.31	*p* < 0.01
VFQ: Colour Vision	-3.32	0.73	-4.55	*p* < 0.01	-18.74	1.34	-13.95	*p* < 0.01
VFQ: Peripheral Vision	-6.91	0.98	-7.04	*p* < 0.01	-25.98	1.80	-14.41	*p* < 0.01
VFQ: Composite Score	-7.05	0.59	-11.91	*p* < 0.01	-23.59	1.10	-21.47	*p* < 0.01

*Notes*: Coefficients were from mixed-effects ordinary least squares regressions of best-corrected visual acuity letter scores, EQ-5D utility, and Visual Function Questionnaire-25 subscale and composite scores on categorical vision bolt-on levels. For instance, comparing with participants who reported “no problem” with their vision, mean BCVA in the worse-seeing eye was 5.23 letters lower among those reporting “some problems” and 13.24 letters lower among those reporting “extreme problems”. Models adjusted for sex at birth, baseline age, and days on trial affected by the COVID-19 pandemic and allowed for a random intercept for each participant. The better-seeing eye was defined as the eye with the higher letter score at the time of measurement; the worse-seeing eye was the one with the lower score. Abbreviations: BCVA, best-corrected visual acuity; CI, confidence interval; SE, standard error; VFQ, Visual Function Questionnaire

**Fig. 1:**
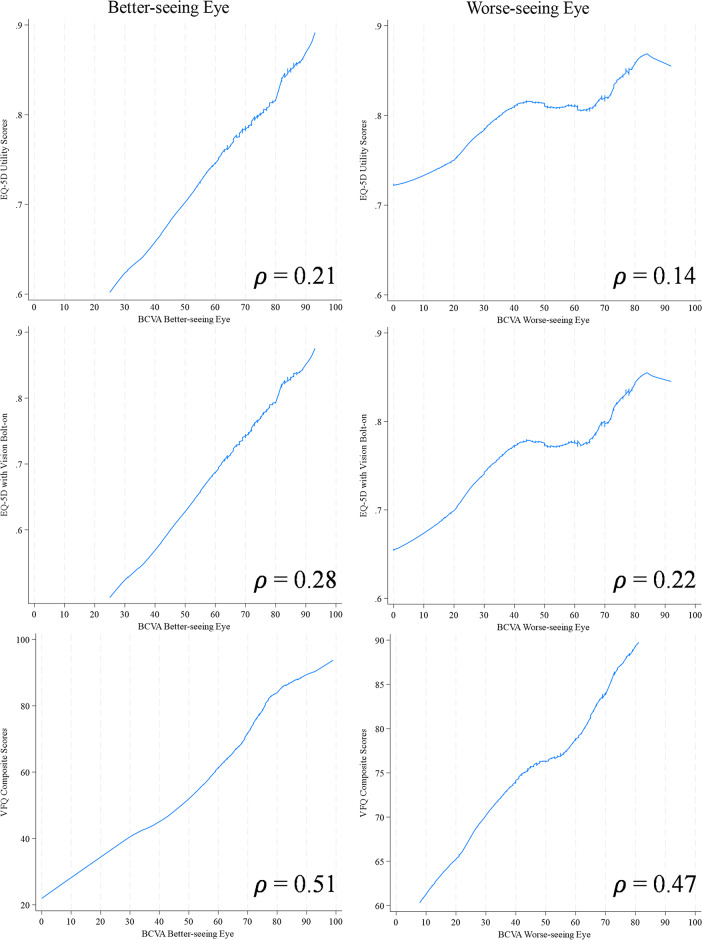
Association between health-related quality of life and visual acuity (BCVA letter scores). Footnotes: Each panel presents the locally weighted scatterplot smoothing between best-corrected visual acuity (BCVA, x-axis) and patient-reported outcomes (y-axis), based on pooled available-case data. Pairwise Spearman rank correlation coefficients (ρ) were reported to capture the monotonic association between best-corrected visual acuity and patient-reported outcomes without assuming linearity, with higher values indicating a stronger positive association. The better-seeing eye was defined as the eye with the higher BCVA letter score between the two eyes for each participant at the time of measurement; the worse-seeing eye was the one with the lower score. Abbreviations: BCVA, best-corrected visual acuity; VFQ, Visual Function Questionnaire

EQ-5D utility scores (with and without the vision bolt-on) and VFQ-25 composite scores increased with BCVA letter scores in the better-seeing eye (column 1; Fig. 1). In the worse-seeing eye (column 2), a plateau was observed between approximately 45–65 letters for EQ-5D utility scores and 45–55 letters for VFQ composite scores. For example, EQ-5D utility scores rose with increasing BCVA up to around 45 letters, flattening between 45 and 65 letters, and showing a modest increase beyond 65 letters (row 1, column 2). We observed stronger correlations between BCVA and quality of life measures in the better-seeing eye than the worse-seeing eye: for EQ-5D utility scores, ρ = 0.21 (better-seeing) vs. 0.14 (worse-seeing); for EQ-5D with vision bolt-on, ρ = 0.28 vs. 0.22; and for VFQ-25 composite scores, ρ = 0.51 vs. 0.47. BCVA was most highly correlated with VFQ-25 and showed a stronger association with EQ-5D with the bolt-on than without (Fig. 1).

**Fig. 2:**
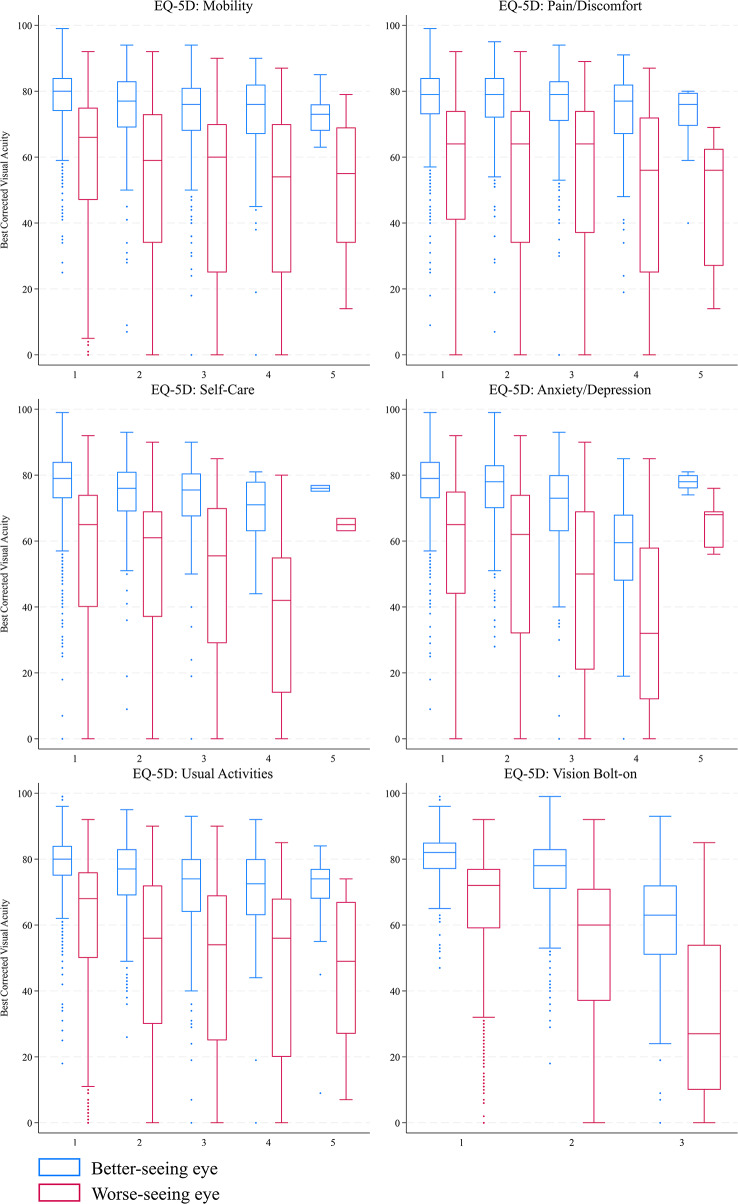
Visual acuity (BCVA letter scores) across EQ-5D response levels. Footnotes: Each panel presents box plots of best-corrected visual acuity (BCVA; y-axis) for the better- and worse-seeing eye across EQ-5D response levels (x-axis), based on pooled available-case data. EQ-5D dimensions are shown at five levels (no problems, slight problems, moderate problems, severe problems, extreme problems). The central line marks the median (50th percentile) BCVA letter score for each EQ-5D; the box spans the range between 75th and 25th quartiles (interquartile); whiskers extend to the values within 1.5 times the interquartile range; and points indicate observations beyond the whiskers. The better-seeing eye was defined as the eye with the higher BCVA letter score between the two eyes for each participant at the time of measurement; the worse-seeing eye was the one with the lower score. Abbreviations: BCVA, best-corrected visual acuity

Figure 2 plots BCVA across EQ-5D dimension levels for the better- and worse-seeing eyes. As problem/difficulty levels increase, median BCVA declines and the distributions shift downward (poorer acuity), with the worse-seeing eye showing wider interquartile ranges.

### Differences in vision-related quality of life between treatment groups

We observed no clinically-meaningful differences between randomised trial groups (SRT vs. sham) at any time point for VFQ subscales, EQ-5D health utilities (with or without the vision bolt-on), or QALYs (with or without the vision bolt-on). This was supported by regression p-values and by the very small Cohen’s d effect sizes (Table 2; Table S4-5). Adding the vision bolt-on slightly increased EQ-5D effect sizes, but the absolute magnitudes remained very small. VFQ subscales showed larger effects, yet generally stayed below the ‘small’ threshold (0.2).

**Table 2:** EQ-5D health utilities with and without vision bolt-on in anti-VEGF alone and SRT plus anti-VEGF groups

	Anti-VEGF alone (*n* = 137)	SRT plus anti-VEGF (*n* = 274)	Difference (SRT plus anti-VEGF minus anti-VEGF alone)^a^		Cohen’s d^b^
	Unadjusted mean (SD)	Unadjusted mean (SD)	Adjusted mean (95% CI)	*p*-value	
**EQ-5D health utilities**					
Baseline	0.83 (0.17)	0.84 (0.19)			
Week 48	0.81 (0.20)	0.82 (0.20)	0.00 (-0.03, 0.03)	0.95	0.01
Week 96	0.77 (0.27)	0.77 (0.27)	0.00 (-0.04, 0.05)	0.92	0.01
Week 144	0.73 (0.29)	0.74 (0.30)	0.01 (-0.05, 0.06)	0.81	0.03
Week 192	0.72 (0.28)	0.73 (0.30)	0.00 (-0.05, 0.06)	0.86	0.02
**EQ-5D health utilities with vision bolt-on (available cases)**					
Baseline (*N* = 411)	0.80 (0.17)	0.81 (0.19)			
Week 48 (*N* = 375)	0.78 (0.20)	0.79 (0.20)	0.00 (-0.03, 0.03)	0.83	0.03
Week 96 (*N* = 371)	0.75 (0.27)	0.76 (0.27)	0.00 (-0.04, 0.05)	0.92	0.01
Week 144 (*N* = 358)	0.72 (0.29)	0.72 (0.30)	0.01 (-0.05, 0.06)	0.75	0.05
Week 192 (*N* = 347)	0.70 (0.28)	0.70 (0.30)	0.00 (-0.05, 0.05)	0.92	0.02

*Notes*: EQ-5D at each time point and QALYs in each year, with missing data imputed using multiple imputation. ^**a**^ Differences and p-values derived from the ordinary least squares regression model adjusted for sex at birth, baseline age, baseline EQ-5D health utility, days on-trial affected by COVID-19 pandemic, and study eye best-corrected visual acuity. ^**b**^ Calculated using available cases. An effect size based on Cohen’s d is typically interpreted as small when between 0.2 and 0.5, moderate when between 0.5 and 0.8, and large when greater than 0.8 [31]. EQ-5D utility scores with the bolt-on are slightly lower than the those without the bolt on as the bolt-on utilities were estimated by subtracting an additional coefficient using published tariff adjustments [18]. Abbreviations: CI, confidence interval; QALY, quality-adjusted life year; SD, standard deviation; SRT, stereotactic radiotherapy; VEGF, vascular endothelial growth factor

### Resource use, costs and cost-effectiveness analysis

At Year 2, participants treated with SRT plus anti-VEGF required significantly fewer injections compared to those receiving anti-VEGF alone (Figure S1). Minimal differences in the number of anti-VEGF injections were observed in Years 3 and 4 where participants returned to routine care and received anti-VEGF therapy according to local drug selection and retreatment criteria.

In Table 3, we report mean values with 95% confidence intervals shown in parentheses. The cost of initial treatment with SRT and the first ranibizumab injection was £1 179 higher per participant in the SRT plus anti-VEGF group than in the anti-VEGF alone group. At Year 2, the mean cost of ranibizumab and its administration was £1 660 lower in the SRT plus anti-VEGF group (95% CI -£2 508 to -£805, *p* < 0.001) due to fewer anti-VEGF injections. The mean costs of monitoring consultations (*p* = 0.68), hospital care (*p* = 0.12) and primary care (*p* = 0.79) did not differ significantly between groups. By Year 2, total costs, including SRT and the first ranibizumab injection, following nAMD management as well as the hospital and primary care costs previously excluded from our preliminary analysis [7, 8] were £464 lower per participant in the SRT plus anti-VEGF group (95% CI: -£1 344 to £420); this difference was not statistically significant (*p* = 0.29).

During Years 3 and 4 (with routine care), the two groups received approximately the same number of anti-VEGF injections, but a slightly greater proportion of participants in the SRT plus anti-VEGF group switched from ranibizumab to aflibercept, which is more expensive: 9.2% (23/251) participants compared to 6.6% (8/137) in the anti-VEGF alone group (Table S9). Consequently, the mean cost of anti-VEGF and administrations was £122 higher in the SRT plus anti-VEGF group (95% CI -£214 to £143, *p* = 0.78). There were negligible differences between groups in the mean costs of monitoring consultations (*p* = 0.73). The mean primary care and medication costs were £46 lower in the SRT group (95% CI -£141 to £1, *p* = 0.19): largely due to one outlier in the anti-VEGF alone group with substantial concomitant medication costs. There were no significant differences between groups in hospital care costs (*p* = 0.46) or total costs (*p* = 0.89).

Over the study period (Years 1–4), participants randomised to the SRT plus anti-VEGF group incurred a mean total cost that was £404 lower per participant than those in the anti-VEGF alone group (95% CI -£2 092 to £1 282), although this difference was not statistically significant (*p* = 0.66).

**Table 3:** Comparison of mean costs and QALYs in anti-VEGF alone and SRT plus anti-VEGF groups

	Anti-VEGF alone (*n* = 137)	SRT plus anti-VEGF (*n* = 274)	Difference (SRT plus anti-VEGF minus anti-VEGF alone)	
	Mean (95% CI)	Mean (95% CI)	Mean (95% CI)	*p*-value
**Years 1–2**				
SRT and the first ranibizumab injection	£715^a^	£1 894^a^	£1 179^a^	
Ranibizumab and administration	£9 288 (£8 536, £10 070)	£7 628 (£6 943, £8 308)	-£1 660 (-£2 508, -£805)	< 0.001
Monitoring consultations	£3 507 (£3 405, £3 601)	£3,527 (£3 447, £3 600)	£20 (-£70, £113)	0.68
Primary care and concomitant medication^b^	£35 (£17, £64)	£21 (£12, £32)	-£14 (-£36, £2)	0.12
Hospital care	£170 (£87, £260)	£181 (£110, £256)	£11 (-£79, £95)	0.79
**Total costs: Years 1–2**	**£13 725 (£12 941**,** £14 554)**	**£13 261 (£12 559**,** £13 968)**	**-£464 (-£1 344**,** £420)**	0.29
**QALYs: Years 1–2**	**1.57 (1.54**,** 1.61)**	**1.57 (1.54**,** 1.61)**	**0.00 (-0.04**,** 0.04)**	0.95
**Years 3–4**				
Anti-VEGF and administration	£5 465 (£4 464, £6 461)	£5 587 (£4 703, £6 461)	£122 (-£786, £1 033)	0.78
Monitoring consultations	£1 709 (£1 510, £1 907)	£1 675 (£1 498, £1 847)	-£34 (-£214, £143)	0.73
Primary care and concomitant medication^b^	£82 (£2, £245)	£36 (£0, £111)	-£46 (-£141, £1)	0.19
Hospital care	£53 (£5, £113)	£71 (£23, £127)	£18 (-£34, £65)	0.46
**Total costs: Years 3–4**	**£7 309 (£6 114**,** £8 498)**	**£7 369 (£6 336**,** £8 390)**	**£60 (-£1 004**,** £1 136)**	0.89
**QALYs: Years 3–4**	**1.30 (1.21**,** 1.39)**	**1.31 (1.23**,** 1.39)**	**0.01 (-0.07**,** 0.09)**	0.84
**Total costs: Years 1–4**	**£21 034 (£19 402**,** £22 695)**	**£20 630 (£19 166**,** £22 079)**	**-£404 (-£2 092**,** £1 282)**	0.91
**Total QALYs: Years 1–4**	**2.876 (2.767**,** 2.979)**	**2.883 (2.781**,** 2.977)**	**0.01 (-0.10**,** 0.11)**	0.66

Note: Results were analysed on an intention-to-treat principle with missing data imputed from multiple imputations and included all participants randomised. Results in this table therefore differ from the complete-case analyses [7, 8]. All costs were originally reported in GBP for the financial year 2021-22. ^a^ Confidence intervals are not shown, since the only variation between participants resulted from four participants in the anti-VEGF alone group who did not receive SRT. Costs and QALYs beyond year one were discounted at 3.5% per year following the NICE reference case [17]. ^b^ Among concomitant medications in the Medical Dictionary for Regulatory Activities organ categories specified a priori as having a plausible relationship to the study intervention (cardiac disorders, eye disorders, neoplasms, and nervous system disorder including stroke), the number of medications (excluding anti-VEGF drugs used for eye disease) started in the first two years of the randomisation differed significantly across treatment groups at the 90% confidence level only in the nervous system disorder category including stroke (*p* = 0.08). Outcomes for the anti-VEGF alone group are from those randomised to sham SRT [7, 8]. CIs were estimated using bootstrapping; CIs and p-values were adjusted for sex at birth, days on-trial during the COVID-19 pandemic, baseline age, baseline EQ-5D health utility, and baseline study eye best-corrected visual acuity. Mean values in each treatment group are presented for the average participant (a woman aged 78 years, who had zero days affected by the COVID pandemic with best-corrected visual acuity of 69 letters and EQ-5D health utility of 0.84). Abbreviations: CI, confidence interval; QALY, quality-adjusted life year; SRT, stereotactic radiotherapy; VEGF, vascular endothelial growth factor

**Fig. 3:**
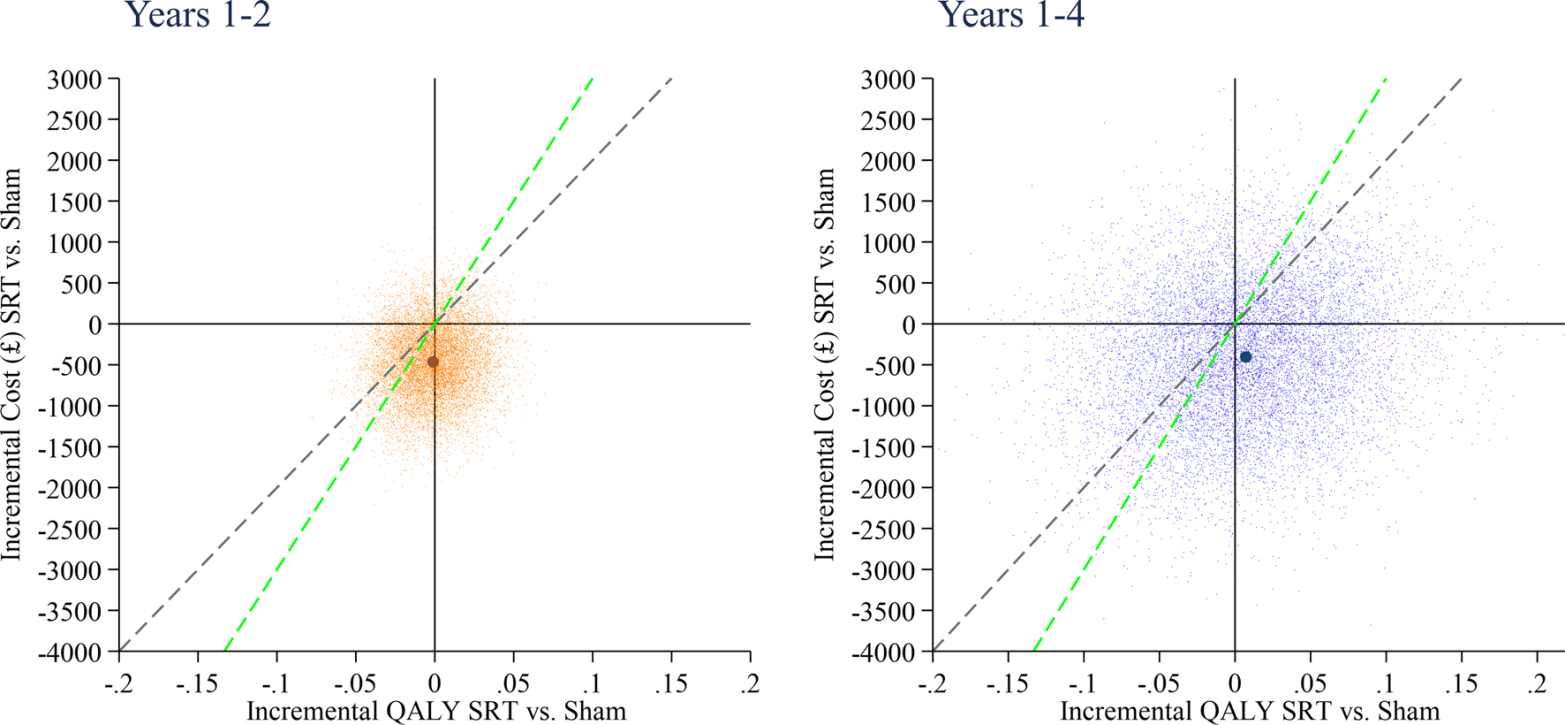
Scatter graphs of Years 1–2 and Years 1–4 evaluation results on the cost-effectiveness plane. Footnotes: The larger orange and blue dot represents the base case cost-effectiveness of SRT plus anti-VEGF against anti-VEGF alone among participants randomised as per the intention-to-treat principle (*n* = 411) with missing data imputed from multivariate imputation for 1–2 years and 3–4 years, respectively. The smaller orange and blue dots represent estimations from non-parametric bootstrapping for1-2 years and 1–4 years, respectively. The grey and green dashed lines are the cost-effectiveness threshold of £20,000/QALY and £30,000/QALY, representing the maximum amount a healthcare system is willing to pay (save) for a unit of health outcome gains (losses). The proportion of bootstrap dots that lie below or to the right of the diagonal lines represents the probability that SRT plus anti-VEGF is cost-effective over anti-VEGF alone

SRT plus anti-VEGF therapy was non-significantly less costly, and participants accrued similar numbers of QALYs over the four-year trial period (Fig. 3). Although there was no significant difference in either costs or effects, we followed best practice in conducting our pre-specified cost-utility analysis and evaluated the cost-effectiveness of SRT and the associated uncertainty, considering the joint distribution of costs and effects [32]. Bootstrapping cost data suggested that there is an 85% probability that SRT plus anti-VEGF costs less than anti-VEGF alone over two years, and 67% over four years (Table S11). At a cost-effectiveness threshold of £20 000 per QALY, the probability of SRT plus anti-VEGF being cost-effective was 78% over two years and 67% over four years (Figure S3). Sensitivity analyses showed that the base case conclusion remained robust in most sensitivity analyses, including changing the discount rate for time preference, excluding concomitant medication costs or including participant transportation costs to a national treatment centre for SRT (Figure S3). Conversely, the cost-effectiveness of SRT plus anti-VEGF was sensitive to the SRT license fee and anti-VEGF price, as well as whether the price of bevacizumab was used in place of ranibizumab. Incorporating the vision bolt-on into the QALY calculation did not alter the overall cost-effectiveness conclusion: SRT remained 77% and 67% likely to be cost-effective at Years 2 and 4, respectively.

## Discussion

Participants reporting vision problems on the EQ-5D bolt-on had worse BCVA, EQ-5D, and VFQ-25 scores than those reporting no vision problems. EQ-5D (with and without vision bolt-on) and VFQ-25 increased more linearly with better-seeing eye BCVA, but showed minimal variation at mid-range BCVA in the worse-seeing eye. The disease-specific VFQ-25 appeared more responsive than EQ-5D (with and without vision bolt-on) across BCVA levels. EQ-5D utilities (with and without the vision bolt-on) did not differ significantly between treatment groups at any time point.

SRT significantly reduced the number of anti-VEGF injections, with cost savings that outweighed the cost of SRT. By Year 4, a one-off SRT treatment reduced direct healthcare costs by £404 per participant, although the total cost difference was not statistically significant. This estimate included nAMD treatment, primary care, hospital care and concomitant medication, and all participants randomised and therefore differed slightly from the results of the simple costing analysis reported previously [7, 8]. Cost-effectiveness analysis suggested that SRT has a 78% probability of being cost-effective if the NHS is willing or able to pay £20 000 per QALY gained. Scaling the cost savings to 174 000 new and 1.1 million existing participants with nAMD in high-income countries [7] suggests that SRT could result in annual savings of £129 million (Table S12). However, the inferior visual acuity observed in the SRT group in Years 3 and 4 [5] means that SRT is unlikely to be a useful addition to nAMD treatment.

This is one of the first studies to evaluate a vision bolt-on item to EQ-5D in a clinical trial setting [11]. Increasing problems with the vision bolt-on were associated with lower BCVA letter scores, EQ-5D domains and utility, and VFQ subscales, indicating that the bolt-on relates to broader vision-related decrements in health status. The difference in utilities estimated with and without the bolt-on was consistent with prior reports of a 0.01–0.03 reduction [12]. The vision bolt-on slightly increased the effect size for between-group differences in EQ-5D, but the effect sizes remained below the threshold for ‘small’ effects. At Year 4, the difference in BCVA between groups was not reflected in EQ-5D or VFQ-25 scores [7, 8], echoing prior findings indicating that EQ-5D performs poorly in macular degeneration [33–35]. Previous studies find that utility scores correlate more with the better-seeing eye than the worse eye among patients with macular degeneration [36, 37], and this was supported by larger correlation coefficients between quality of life and better-seeing eye BCVA as shown in our study. In STAR, only one eye received treatment, and 63% (260/411) had better vision in the non-study eye at baseline. This clinical context may have reduced the perceived impact of BCVA loss and contributed to the plateau seen in Fig. 1, where further deterioration in the worse-seeing eye BCVA had limited influence on quality of life until BCVA fell below approximately 30 letters. This does not mean the worse-seeing eye is unimportant: impairment in the worse-seeing eye independently reduces utility and VFQ-25 scores, especially when binocular function is considered [37–41]. This underscores the importance of presenting BCVA alongside HRQoL outcomes and interpreting outcomes in the context of both eyes.

This is the first economic evaluation of SRT to include a comprehensive assessment of resource use. Sensitivity analyses suggested that the cost-effectiveness of SRT plus anti-VEGF was influenced by the SRT license fee and anti-VEGF pricing. SRT plus anti-VEGF would no longer be cost-effective if the SRT license fee increased by 147% or anti-VEGF costs fell by 42% or more. This may be relevant as biosimilar drugs emerge on the market and as the NHS receives confidential discounts from anti-VEGF manufacturers. Adjunctive treatments like SRT could, nonetheless, ease the ophthalmology clinic burden. Perhaps for this reason, 60.7% (222/372) of participants received a “treat-and-extend” regimen at some point in Years 3 and 4. More participants treated with SRT switched to aflibercept, although the injection frequency over Years 3 and 4 did not differ significantly (9.3 vs. 10.4, respectively, *p* = 0.28). The extended follow-up captured real-world transitions from trial to routine care and revealed longer-term uncertainty in visual and cost outcomes. While repeated SRT could be explored, long-term radiation effects remain unclear, and a single dose already showed small harms [8].

Study limitations include not capturing indirect costs (e.g., low vision support) and relying on participant-reported healthcare use (other than injections), although a one-year recall period may be reasonably reliable for this type of data [42]. Findings are based on a UK clinical setting and may vary by country, health system, and SRT site volume.

In summary, the vision bolt-on captured differences in quality of life between participants but may not be sufficiently responsive to visual acuity decline in trials where only one eye is treated. The cost savings with SRT plus anti-VEGF need to be interpreted cautiously due to long-term visual acuity inferiority, highlighting the critical importance of extended, masked, real-world follow-up in clinical trials. The on-trial and routine care resource use data provide a valuable reference for future economic evaluations.

## Supplementary Information

Below is the link to the electronic supplementary material.

Supplementary Material 1

## Data Availability

All the data generated during this study are included in the manuscript and the supplementary materials.
