# Supplementary material for: EQ-5D vision bolt-on in macular degeneration: associations with visual measures and effect on utility differences and cost-effectiveness of stereotactic radiotherapy
Source: Health Qual Life Outcomes. 2026 Jul 14;24:93. doi: 10.1186/s12955-025-02457-w (PMC13371251; doi:10.1186/s12955-025-02457-w)
Supplement: Supplementary file 1 — Supplementary Material 1 [file 12955_2025_2457_MOESM1_ESM.docx]

**EQ-5D vision bolt-on in macular degeneration: Associations with visual measures and effect on utility differences and cost-effectiveness of stereotactic radiotherapy**

Xuemin Zhu, PhD,^1^ Sarah Wordsworth, PhD,^1,2^ Chan Ning Lee, FRCOphth,^3,4^ Hatem A. Wafa, MPH,^5^ Yanzhong Wang, PhD,^5^ Riti Desai, MPhil,^3,4^ Lisa Ramazzotto, MPharm,^3,4^ Barnaby C Reeves, DPhil,^6^  Timothy L Jackson, FRCOphth,^3,4*^ and Helen Dakin, DPhil^2*^ on behalf of the STAR Study Group

* These authors contributed equally

^1^Health Economics Research Centre, University of Oxford, Oxford, UK

^2^ Oxford NIHR Biomedical Research Centre

^3^Faculty of Life Sciences and Medicine, King’s College London, London, UK

^4^King’s Ophthalmology Research Unit (KORU), King’s College Hospital, London, UK

^5^Population Health Sciences, King’s College London, London, UK

^6^Population Health Sciences, Bristol Medical School, University of Bristol, Bristol, UK

**Contact details for corresponding author:**

**Xuemin Zhu,** Health Economics Research Centre, Nuffield Department of Population Health, University of Oxford, Old Road Campus, Headington, Oxford, OX3 7LF, UK. Email: [xuemin.zhu@ndph.ox.ac.uk](mailto:xuemin.zhu@ndph.ox.ac.uk). Orchid ID: <https://orcid.org/0000-0001-8135-3956>

Supplementary Materials – Index

[STAR Health Economic Analysis Plans (HEAP) for the two-year base case analysis 3](#_Toc209108890)

[Supplementary Methods 8](#_Toc209108891)

[Overview 8](#_Toc209108892)

[Measuring costs 8](#_Toc209108893)

[Table S1: Unit costs 2021 UK £ 9](#_Toc209108894)

[Measuring health outcomes 12](#_Toc209108895)

[Missing data and uncertainty 13](#_Toc209108896)

[Table S2. Multiple imputation model specification 13](#_Toc209108897)

[Cost-utility analysis 15](#_Toc209108898)

[Sensitivity analyses 16](#_Toc209108899)

[Justification for no subgrouping or distributional analysis 17](#_Toc209108900)

[List of assumptions used within the analysis 18](#_Toc209108901)

[Primary care and concomitant medications—Primary care 18](#_Toc209108902)

[Primary care and concomitant medications— Concomitant medications 19](#_Toc209108903)

[Hospital care—consultations and procedures 21](#_Toc209108904)

[Hospital care— Hospitalisation 23](#_Toc209108905)

[EQ-5D Utilities and QALYs 25](#_Toc209108906)

[Year 3/4 –Identifying monitoring regimen for each participant 25](#_Toc209108907)

[Injection and monitoring visit 27](#_Toc209108908)

[Days on-trial during the COVID-19 pandemic 27](#_Toc209108909)

[Multiple imputation 27](#_Toc209108910)

[Supplementary Results 30](#_Toc209108911)

[Table S3. Response levels for vision bolt-on at each time point (available cases) 30](#_Toc209108912)

[Table S4. VFQ subscales at each time point (available cases) 30](#_Toc209108913)

[Table S5. QALY with and without vision bolt-on at each time point 32](#_Toc209108914)

[Table S6: Vision and health-related quality of life across vision bolt-on levels 32](#_Toc209108915)

[Table S7: EQ-5D responses across vision bolt-on levels 33](#_Toc209108916)

[Table S8. Coefficients of vision bolt-on on EQ-5D domains 34](#_Toc209108917)

[Figure S1. Comparison of the number of anti-VEGF injections over time 35](#_Toc209108918)

[Table S9. Number of participants receiving each anti-VEGF drug in years 3 and 4 (available cases) 35](#_Toc209108919)

[Table S10. Comparison of resource use quantities in the anti-VEGF monotherapy and SRT plus anti-VEGF groups 35](#_Toc209108920)

[Figure S2. The probability of SRT plus anti-VEGF being cost-effective compared with anti-VEGF alone at an NHS cost-effectiveness threshold of £20 000 per QALY 36](#_Toc209108921)

[37](#_Toc209108922)

[Figure S3. Cost-effectiveness acceptability curves for two and four years 38](#_Toc209108923)

[Table S11. Results of sensitivity analysis and subgroup analyses for SRT plus anti-VEGF versus anti-VEGF monotherapy 39](#_Toc209108924)

[Table S12. Potential budget impact from SRT for high-income countries globally. Adapted from Jackson et al. (2024) Table S5 ^7^ 42](#_Toc209108925)

[References 44](#_Toc209108926)

# STAR Health Economic Analysis Plans (HEAP) for the two-year base case analysis

- Aim to assess whether 16 Gray Stereotactic Radiotherapy (SRT), in conjunction with ranibizumab, is cost-effective compared with ranibizumab monotherapy.
  - A secondary aim is to compare costs and QALYs between SRT+ranibizumab and ranibizumab alone.
- Base case analysis will take an NHS and personal social services perspective, following NICE reference case.^1^
- Two-year time horizon, matching the primary endpoint.
- Intention-to-treat analysis, including all patients randomised, irrespective of whether they receive the allocated treatment or have complete data. Patients will be analysed in the groups to which they were randomised, irrespective of treatment received.
- Multiple imputation of missing data will be used for the analyses presented in the economic evaluation paper to enable all patients randomised to be included in the analysis, even if they are missing one or more resource use or EQ-5D utility measurement. The number of imputed datasets (M) will equal the percentage of patients with missing data on any resource use or utility measurement during the 24-month time horizon.
- The main outcome measure will be the cost per quality-adjusted life year (QALY) gained for SRT plus ranibizumab versus ranibizumab alone, which will capture quality of life differences between groups. Participants will complete the National Eye Institute 25 Item Visual Function Questionnaire (VFQ-25) and the EuroQoL EQ-5D at enrolment and then yearly.
- EQ-5D-5L will be valued using the Hernandez Alva crosswalk tariff^2^ that forms part of the NICE reference case.^1^
  - QALYs will be estimated from EQ-5D-5L utilities assuming linear changes between EQ-5D-5L measurements. Patients who die will be assumed to have constant utility between the last EQ-5D-5L measurement and the date of death.
- Bootstrapping will be used to quantify uncertainty and plot cost-effectiveness acceptability curves and estimate 95% confidence intervals. B bootstraps will be drawn independently from each of the M imputed datasets (stratified by treatment allocation) and confidence intervals and cost-effectiveness and acceptability curves will be based on the B*M estimates of incremental costs and QALYs.
  - Cost-effectiveness ratios and estimates of incremental QALYs and costs will use linear regression to adjust for age, gender, baseline EQ-5D utility, baseline EDTRS best-corrected visual acuity (BCVA) and the number of months from randomisation to start of COVID restrictions (23^rd^ March 2020), capped at 24 months. This will be done to minimise the risk of bias from chance imbalance between randomised groups. Adjusting for age and gender primarily adjusts for any chance difference in mortality that may result from imbalance in age/gender, while adjusting for baseline EQ-5D utility is an established method to avoid bias in QALY calculations.^3^ We will adjust for the timing of COVID restrictions to overcome any chance imbalance in the number of follow-up appointments and injections that may have been cancelled due to COVID that could otherwise have led to bias.
- Discount costs and QALYs at 3.5% per annum.^1^
- Reference year for costs will be 2021-2.
- Conclusions about whether treatment is cost-effective will be based on a £20,000/QALY ceiling ratio, assuming that the NHS is willing/able to pay up to £20,000 to gain one QALY or is willing to accept the loss of one QALY to save at least £20,000.^1^
- Mean differences in costs and QALYs will be evaluated at the two-sided 0.05 significance level.
- Costing: Costing analyses will focus on resource use related to the study eye.
  - The costing analysis for the main economic evaluation paper will include:
    - The cost of SRT: based on the licence fee plus the staff time included in the grant costings for national treatment centres
    - Monthly check-up visits regarding their study eye (or both eyes) will be included as this is the basis of retreatment decisions. Monitoring consultations with and without intravitreal injection or FFA will be costed using the consultation costs from Department of Health reference costs, following the methods of recent NICE appraisals.^4,5^ No cost will be applied to visits that were scheduled but not attended (regardless of the reason for non-attendance). No cost will therefore be applied to visits that were missed due to the COVID pandemic or lockdown: this approach avoids imposing unnecessary assumptions on the analysis and allows for the fact that patients whose vision has deteriorated are more likely to attend the clinic during a lockdown than patients whose vision has remained good. Visits that were conducted virtually rather than face-to-face will be costed at the same price as face to face monitoring visits.
    - Annual fundus photography and FFA will not be included in the costing as these investigations are not done in routine clinical practice. The cost of one FFA will be applied to each SAE of radiation retinopathy, in the expectation that clinicians would want to confirm and quantify radiation retinopathy. However, we excluded radiation retinopathy reported by attending clinicians at the 12 or 24-month visits, as these were likely to have been detected on the mandated FFAs that occur at these visits, and FFAs would not be undertaken in routine care. Likewise, radiation retinopathy detected by the reading centre will also be excluded, as this would also not occur outside of a trial. The baseline FFA in both arms will also be excluded as all trial participants had received at least 3 anti-VEGF injections before randomisation and if an FFA is required this will usually have been completed before the first injection.
    - Consultations with community health practitioners, hospital consultations and hospital admissions/procedures regarding the study eye. No data were collected on consultations and admissions that were not related to the study eye, or residential care, low-vision aids or personal care in order to minimise the burden on patients and reduce chance variation in resource use.
  - Ranibizumab will be costed as Lucentis (not a biosimilar) in the base case analysis. Since there is no publicly available data on the price paid by the NHS, we will use the list price in the economic evaluation.
  - VAT will be excluded, as per NICE manual.^1^
  - Cost of concomitant medications (other than anti-VEGF) will be excluded from the analysis unless initial analyses find that there is a statistically-significant difference between study arms in the mean number of new concomitant medications started during the first two years of the trial in one of the following MedDRA organ classes that were specified a priori as having a plausible relationship to study interventions: (2) Cardiac disorders; (6) Eye disorders (excluding anti-VEGF); (16) Neoplasms; (17) Nervous system disorder (including stroke). The cost of concomitant medications will be included for any organ classes in which there is a statistically significant difference between groups in a two-tailed t-test with an alpha=0.1 significance level to reduce the risk that important differences in medication use between groups are not missed.
  - Patient transport costs to the national treatment centres will be excluded from the base case analysis on the basis that they would be not normally be funded by the NHS. In a sensitivity analysis including transport costs, these will be valued at the price at which trial centres were reimbursed in the study (£175) adjusted for inflation.
  - No cost will be applied to sham SRT.
  - Other types of resources will not be included in the analysis since they were not anticipated to differ between arms and therefore no data were collected.
  - Since radiotherapy would be expected to affect only the study eye, data were not collected on treatments or consultations related to the fellow eye and all monitoring visits and ranibizumab injections were costed on the basis that only one eye was being treated.
- Sensitivity analyses:
  - Subgroup analysis focusing on patients who had completed two years’ follow-up by 23rd March 2020 (unaffected by COVID).
  - If any of the planned subgroup analyses specified in the statistical analysis plan have a statistically significant interaction with the primary endpoint at the 5% level, we will do subgroup analyses on those variables.
  - Varying the licence fee for SRT between £0 and the base case figure to identify at what price SRT costs £20,000/QALY and at what price SRT and no SRT have equal cost.
  - Assuming that patients have monitoring visits only every 2 months after the 1^st^ year of the trial (with no change in the number of ranibizumab injections).
  - Including the cost of monthly visits that were cancelled/missed – assuming perfect attendance.
  - Applying unit cost of bevacizumab rather than ranibizumab using the price from the IVAN trial (£49/dose^6^), adjusted for health care inflation; this price has also been used in two recent NICE appraisals.^4,5^ We will also do an analysis reducing the price of Lucentis by 20%, 30%, 50% and 80% and do an analysis using unit price per dose of aflibercept (Eylea) but not changing the dosing frequency. If the biosimilar ranibizumab becomes available before the economic evaluation is completed, we would also do a sensitivity analysis using that price.
  - One-way sensitivity analysis varying the cost of SRT.
  - Including patient transport costs at £175/patient (plus inflation from the median year of recruitment to 2022 values).
  - Utilities using the vision bolt-on to EQ-5D.

# Supplementary Methods

## Overview

The cost-effectiveness analysis was based on the STAR study.^7^ The primary analysis compared the cost-utility of stereotactic radiotherapy (SRT) plus ranibizumab against anti-VEGF monotherapy over two years from the perspective of the UK National Health Service (NHS). Our analysis also extended the observation period to four years, allowing participants to switch to other anti-VEGF drugs and other monitoring and retreatment regimens.

We included direct healthcare costs for the study eye. All costs were referenced to the year 2021/22.^8-12^ Health outcomes were measured in the form of quality-adjusted life years (QALYs) by aggregating EQ-5D-5L utilities, assuming a linear change between annually observed time points.^13^ Resource use data were gathered through trial case report forms completed by a nurse on behalf of the participant. Costs and QALYs were discounted at an annual rate of 3.5%.^1^

## Measuring costs

The base case analysis adopted an NHS perspective following NICE guidance.^1^ All resource use associated with the study eye was recorded on standard trial forms. At specified study visits (every 4 weeks for the first 96 weeks, with additional visits at weeks 144 and 196), participants reported hospital admissions and medical professional contacts related to the study eye and all medication.

The costing analysis encompassed nAMD treatment and direct healthcare resource utilization related to the study eye, including SRT, monitoring consultations, anti-VEGF administrations, primary care and concomitant medications, and hospital care. Non-treatment-related resource use that was not attributable to the study eye or intervention was excluded to isolate the impact of treatment.^14^ Concomitant medication costs were included based on pre-specified criteria in the analysis plan, focusing on MedDRA organ categories with plausible links to the study intervention and statistically significant differences in new prescriptions during the first two years (see below).

NHS costs per participant were computed by multiplying the reported frequency of each NHS resource by its unit cost across all relevant categories (Table S1). SRT costs were estimated through microcosting methods reported previously,^7^ while unit costs for hospital care and primary care were sourced from the NHS Reference Costs and PSSRU Unit Cost compendia.^8,15^ Medication costs related to the study eye were based on the British National Formulary or prescription cost analysis, assuming generic dosage forms.^10,11^ All costs were expressed in 2021/22 UK pounds sterling, adjusted where necessary to reflect 2021/22 prices using the NHS cost inflation pay and prices index.^8^

### Table S1: Unit costs 2021 UK £

| **Resource (units)** | **Cost (£)** | **Source** |
| --- | --- | --- |
| **SRT and ranibizumab administration** | | |
| Monitoring consultation | 159.05 | Outpatient procedure code for retinal tomography (ophthalmology) 19 years and over BZ88A in National Schedule of NHS Costs Year 2021-22 Outpatient procedures. ^8^ |
| Injection consultation | 157.39 | A weighted average of two procedure codes (weighting by number of examinations in England): BZ86B Intermediate Vitreous Retinal Procedures, 19 years and over, with CC Score 0-1 (Ophthalmology) and BZ87A Minor Vitreous Retinal Procedures, 19 years and over (Ophthalmology). National Schedule of NHS Costs Year 2021-22 Outpatient procedures. ^8^ |
| Ranibizumab (per 0.5-mg dose) | 551.00 | British National Formulary. ^10^ Cost per vial or pre-filled disposable injection containing ranibizumab (Lucentis) 10 mg/mL solution for injection. |
| SRT | 1 342.91 | Micro-costing from the STAR study. ^7^ |
| **Primary care** | | |
| GP (per surgery consultation lasting 9.22 minutes) | 38.00 | PSSRU 2022,^8^ 66 pp. 9.4.2: Unit costs for a GP. Includes qualifications, but excludes direct staff costs |
| GP home visit | 86.84 | PSSRU 2022,^8^ 66 pp. 9.4.2: Unit costs for a GP. Includes qualifications, but excludes direct staff costs. Assume the home visit same duration/cost as the surgery visit, and 12 minutes of travel time^16^ |
| GP telephone consultation (per phone call) | 15.80 | PSSRU 2022,^8^ 69 pp. 9.6.1: Costs and unit estimations for a telephone triage. Cost per intervention (including other costs) |
| GP Nurse (per surgery consultation) | 17.46 | PSSRU 2022,^8^ 64 pp. 9.3.1: Costs and unit estimations for nurses working in a GP practice nurse. £52 per hour, including qualification costs. The ratio of direct-to-indirect time on face-to-face contacts is 1 : 0.30, and the average contact time is 15.5 minutes^16^ |
| GP nurse home visit (per consultation) | 30.98 | PSSRU 2022,^8^ 64 pp. 9.3.1: Costs and unit estimations for nurses working in a GP practice nurse. £52 per hour, including qualification costs. The ratio of direct-to-indirect time on face-to-face contacts is 1: 0.30, and the average contact time is 15.5 minutes. Assume the home visit same duration/cost as the surgery visit, and 12 minutes of travel time^16^ |
| General practice nurse telephone consultation (per phone call) | 8.69 | PSSRU 2022,^8^ 69 pp. 9.6.1: Costs and unit estimations for a telephone triage. Cost per intervention (including other costs) |
| District nurse home visit (per consultation) | 53.74 | National schedule of NHS costs 2021/22. Community health services - nursing, N02AF, District Nurse, Adult, Face to face |
| Calls to NHS 111 (formally NHS Direct) | 15.49 | The Financial Times^17^ inflated to 2021/22 values using NHS cost inflation pay and prices index^8^ |
|  |  |  |
| **Hospital consultations** | | |
| Plastic Surgery Service (per consultation) | 159.89 | National schedule of NHS costs 2021/22.^15^ Total outpatient attendance - 115, First outpatient appointment |
| Neurology Service (per consultation) | 194.08 | National schedule of NHS costs 2021/22.^15^ Total outpatient attendance - 150, Follow-up outpatient appointment |
| Neurology Service (per consultation) | 263.32 | National schedule of NHS costs 2021/22.^15^ Total outpatient attendance - 150, First outpatient appointment |
| Emergency Medicine Service (per consultation) | 144.00 | National schedule of NHS costs 2021/22.^15^ Total outpatient attendance - 180, Total |
| Medical Ophthalmology Service (per consultation) | 125.92 | National schedule of NHS costs 2021/22.^15^ Outpatient care - 460, Total |
| Medical Ophthalmology Service (per consultation) | 169.38 | National schedule of NHS costs 2021/22.^15^ Outpatient care - 460, First outpatient appointment |
| Medical Ophthalmology Service (per consultation) | 117.45 | National schedule of NHS costs 2021/22.^15^ Emergency care - Weighted averaged across VB04Z, VB05Z, VB06Z, VB07Z, VB08Z, VB09Z, and VB11Z |
| Medical Ophthalmology Service (per consultation) | 123.71 | National schedule of NHS costs 2021/22.^15^ Outpatient care - 460, Follow-up outpatient appointment |
| Orthoptics Service (per consultation) | 107.21 | National schedule of NHS costs 2021/22.^15^ Outpatient care - 655, First outpatient appointment |
| Orthoptics Service (per consultation) | 105.58 | National schedule of NHS costs 2021/22.^15^ Outpatient care - 655, Follow-up outpatient appointment |
| Diagnostic imaging service (per consultation) | 46.00 | National schedule of NHS costs 2021/22.^15^ Total outpatient attendance - 812, Total |
| NHS walk-in centers (per consultation) | 81.93 | National schedule of NHS costs 2021/22.^15^ Emergency care - Weighted averaged across VB04Z, VB05Z, VB06Z, VB07Z, VB08Z, VB09Z, VB11Z |
|  |  |  |
| **Outpatient procedures** | | |
| Phacoemulsification Cataract Extraction and Lens Implant (per attendance) | 1287.01 | National schedule of NHS costs 2021/22.^15^ Admitted Patient Care - daycase, Weighted average cost across BZ34A, BZ34B, BZ34C |
| YAG Laser Capsulotomy (per attendance) | 181.04 | National schedule of NHS costs 2021/22.^15^ Admitted Patient Care - outpatient, BZ33Z |
| YAG Laser Capsulotomy (per attendance) | 206.81 | National schedule of NHS costs 2021/22.^15^ Admitted Patient Care - daycase, BZ33Z |
| Pars Plana Vitrectomy (per attendance) | 203.60 | National schedule of NHS costs 2021/22.^15^ Admitted Patient Care - outpatient, BZ84B |
| Pars Plana Vitrectomy (per attendance) | 628.66 | National schedule of NHS costs 2021/22.^15^ Admitted Patient Care - daycase, BZ84B |
| Vitreous Biopsy and Intravitreal Antibiotics (per attendance) | 152.69 | National schedule of NHS costs 2021/22.^15^ Admitted Patient Care - outpatient BZ86B |
| Vitreous Biopsy and Intravitreal Antibiotics (per attendance) | 328.17 | National schedule of NHS costs 2021/22.^15^ Admitted Patient Care - daycase, BZ86B |
| Lensectomy (per attendance) | 1706.10 | National schedule of NHS costs 2021/22.^15^ Admitted Patient Care - daycase, BZ32A |
| Retinal repair (per attendance) | 328.17 | National schedule of NHS costs 2021/22.^15^ Admitted Patient Care - total, BZ86B |
| Biopsy of Temporal Artery (per attendance) | 1747.98 | National schedule of NHS costs 2021/22.^15^ Admitted Patient Care - daycase YQ43Z |
| Endolaser dacryocystorhinostomy (per attendance) | 2,538.63 | National schedule of NHS costs 2021/22.^15^ Admitted Patient Care – daycase, Weighted averaged across BZ54A and BZ54B |
| Laser iridotomy (per attendance) | 215.34 | National schedule of NHS costs 2021/22.^15^ Admitted Patient Care - total BZ94B |
|  |  |  |
| **Hospitalizations** |  |  |
| Minor Oculoplastics Procedures, 19 years and over (per attendance) | 1651.98 | National schedule of NHS costs 2021/22.^15^ Admitted Patient Care - inpatients, Weighted average cost across elective inpatient and non-elective inpatient BZ46A |
| Non-surgical Ophthalmology with length of stay 2 days or more (per attendance) | 1411.37 | National schedule of NHS costs 2021/22.^15^ Admitted Patient Care - inpatients, Weighted average cost across elective inpatient and non-elective inpatient BZ24D, BZ24E, BZ24F, BZ24G |
| Non-surgical Ophthalmology with length of stay 1 day or less (per attendance) | 590.79 | National schedule of NHS costs 2021/22.^15^ Admitted Patient Care - daycase, Weighted average cost across BZ24D, BZ24E, BZ24F, BZ24G |
|  |  |  |
| **Other anti-VEGF drugs** |  |  |
| Faricimab (Vabysmo) | £857.00 | British National Formulary.^10^ Cost per pre-filled syringes containing Faricimab (Vabysmo) 120mg/ml solution for injection. |
| Bevacizumab (Avastin) (per 1.25 mg dose) | 58.41 | Micro-costing from the IVAN study ^18^, also used in recent NICE appraisals ^4,5^, is based on manual repackaging and dilution of bevacizumab (Avastin) 25 mg/mL solution. Medicine cost is from the British National Formulary, with 28% attributed to ingredients. Non-medicine costs are inflated using the Personal Social Services Pay & Prices Index ^8^. |
| Aflibercept (Eylea) | £816.00 | British National Formulary.^10^  Cost per vial or pre-filled disposable injection containing Aflibercept (Eylea) 40 mg/mL solution for injection.^9^ |
| *Notes:* Unit costs for SRT and ranibizumab administration were adapted from Jackson et al. (2024) Table S5 ^7^. *All costs were originally reported in GBP for the financial year 2021-22. Abbreviations: NHS, national health service; SRT, stereotactic radiotherapy. GP, general practitioner; PSSRU, Personal Social Services Research Unit; NHS, National Health Service; NICE, National Institute for Health and Clinical Excellence | | |

## Measuring health outcomes

The health-related quality of life of trial participants was measured at baseline and at one, two, three, and four years after randomisation. The EuroQol five-dimension five-level questionnaire (EQ-5D-5L) was used as the primary health outcome measure as a standardized instrument that allows for comparisons across different disease areas and populations within a healthcare system.^19^ Additionally, a vertical visual analogue scale (VAS) that records the participant’s self-rated health was included to provide a more comprehensive assessment of overall health status.^19^

Following NICE recommendation^1^, the EQ-5D utilities were cross-walled from five-level to three-level based on age.^13^ Two-year QALYs ($\mathrm{QALYs}_{Y2}$) for each participant were estimated by using the EQ-5D-5L utility to calculate the area under the curve across the three utility (*Utility*) measures, assuming linear changes between each measurement. Likewise for four-year QALYs.

$$\mathrm{QALYs}_{Y2}=\frac{{Utility}_{baseline}+{Utility}_{Y1}}{2}*\frac{\mathrm{DayLived}_{Y1}}{336}$$

$$+\frac{{Utility}_{Y1}+{Utility}_{Y2}}{2}*\frac{\mathrm{DayLived}_{Y2}}{336}$$

Following the established methods to avoid the chance of baseline imbalance,^3^ mean QALYs were estimated by regressing QALYs on treatment allocation, baseline EQ-5D utility, age, gender, baseline EDTRS BCVA, days on-trial during the COVID-19 pandemic.

A vision ‘bolt-on’ item was appended to the EQ-5D-5L questionnaire to capture the potential impact of visual impairment on quality of life.^20^ Based on the scoring algorithm for the vision bolt-on for EQ-5D-3L,^20^ a decrement of 0.0378 was subtracted from the EQ-5D utility score for participants who reported “some problems” with vision and a decrement of 0.130 was subtracted from the EQ-5D-3L score for participants who report “extreme problems” with vision. Constant and N3 terms were not added to avoid giving undue weight to the vision bolt-on in situations where the participant has judged that the vision does not affect other domains on the EQ-5D.

## Missing data and uncertainty

To handle missing data in cost and utility, we utilized Multiple Imputation by Chained Equations (MICE), a principled method of dealing with missing data.^21^ Through iterative imputation cycles, each missing value was estimated using variables as outlined in Table S2. We used 37 imputations, based on the proportion of participants with any missing data over the four years (37%).

We afterwards aggregated yearly costs from quarterly components. Each imputed dataset was independently bootstrapped 1000 times, resulting in 37,000 sets of results. Mean costs, QALYs, and other sensitivity analysis-defined results were estimated for each bootstrap on each imputed dataset. The 37,000 sets of bootstrapped results were pooled together, which is equivalent to Rubin’s rule ^22,23^ and addresses uncertainty around estimated coefficients. We calculated 95% confidence intervals using the 2.5th and 97.5th percentiles across the 37,000 bootstraps.

### Table S2. Multiple imputation model specification

| **Variables** | **Imputation function** | **Explanatory variables** | **Rationale** |
| --- | --- | --- | --- |
| Age | N/A | N/A | Baseline covariates |
| Gender | N/A | N/A | Baseline covariates |
| Treatment allocation | N/A | N/A | Treatment indicator |
| Baseline EQ-5D health utility | Single mean imputation | N/A | Strong predictor of later health state and independent of baseline covariates |
| Baseline vision bolt-on EQ-5D health utility | Single mean imputation | N/A | Strong predictor of later health state and independent of baseline covariates |
| Baseline study eye EDTRS BCVA | Single mean imputation | N/A | Strong predictor of health outcome and related resource use, independent of baseline covariates |
| Baseline fellow eye EDTRS BCVA | Single mean imputation | N/A | Strong predictor of health outcome and related resource use, independent of baseline covariates |
| Year 1-4: study eye EDTRS BCVA | Regress, pmm | Full model, conditional on participant alive in the last quarter | Strong predictors of health outcome and related resource use |
| Year 1-4: fellow eye EDTRS BCVA | Regress, pmm | Full model, conditional on participant alive in the last quarter | Strong predictors of health outcome and related resource use |
| Quarter 1-8: Being alive | N/A | N/A | Conditions for multiple imputation |
| Year 3: Being alive | N/A | N/A | Condition for multiple imputation |
| **Imputation model for 2-year cost-utility analysis** | | | |
| Quarter 1-8: Number of monitoring visits | Regress, pmm | Full model, conditional on participant alive in the last quarter | Cost components |
| Quarter 1-8: Number of ranibizumab administration | Regress, pmm | Full model, conditional on participant alive in the last quarter | Cost components |
| Quarter 1-8: Cost of primary care | Regress, pmm | Full model, conditional on participant alive in the last quarter | Cost components |
| Quarter 1-8: Cost of hospital care | Regress, pmm | Full model, conditional on participant alive in the last quarter | Cost components |
| Year 1 Cost of new concomitant medicines | Regress, pmm | Full model, conditional on participant alive in the last quarter | Cost component |
| Year 2 Cost of new concomitant medicines | Regress, pmm | Full model, conditional on participant alive in the last quarter | Cost component |
| Year 1 EQ-5D-5L health utility | Regress, pmm | Full model, conditional on participant alive in the last quarter | Health outcome |
| Year 2 EQ-5D-5L health utility | Regress, pmm | Full model, conditional on participant alive in the last quarter | Health outcome |
| Year 1 vision bolt-on EQ-5D utility | Regress, pmm | Full model, conditional on participant alive in the last quarter | Health outcome sensitivity analyses |
| Year 2 vision bolt-on EQ-5D utility | Regress, pmm | Full model, conditional on participant alive in the last quarter | Health outcome sensitivity analyses |
| **Imputation model for 4-year cost-utility analysis** | | | |
| Year 3 Costs of monitoring visit | Regress, pmm | Full model, conditional on participant alive in the last quarter | Cost component |
| Year 4 Costs of monitoring visit | Regress, pmm | Full model, conditional on participant alive in the last quarter | Cost component |
| Year 3 Number of anti-VEGF administration | Regress, pmm | Full model, conditional on participant alive in the last quarter | Cost component and primary clinical outcome |
| Year 4 Number of anti-VEGF administration | Regress, pmm | Full model, conditional on participant alive in the last quarter | Cost component and primary clinical outcome |
| Year 3 Costs of anti-VEGF administration | Regress, pmm | Full model, conditional on participant alive in the last quarter | Cost component |
| Year 4 Costs of anti-VEGF administration | Regress, pmm | Full model, conditional on participant alive in the last quarter | Cost component |
| Year 3 Cost of primary care | Regress, pmm | Full model, conditional on participant alive in the last quarter | Cost component |
| Year 4 Cost of primary care | Regress, pmm | Full model, conditional on participant alive in the last quarter | Cost component |
| Year 3 Cost of hospital care | Regress, pmm | Full model, conditional on participant alive in the last quarter | Cost component |
| Year 4 Cost of hospital care | Regress, pmm | Full model, conditional on participant alive in the last quarter | Cost component |
| Year 3 Cost of new concomitant medicines | Regress, pmm | Full model, conditional on participant alive in the last quarter | Cost component |
| Year 4 Cost of new concomitant medicines | Regress, pmm | Full model, conditional on participant alive in the last quarter | Cost component |
| Year 3 EQ-5D-5L health utility | Regress, pmm | Full model, conditional on participant alive in the last quarter | Health outcome |
| Year 4 EQ-5D-5L health utility | Regress, pmm | Full model, conditional on participant alive in the last quarter | Health outcome |
| Year 3 vision bolt-on EQ-5D utility | Regress, pmm | Full model, conditional on participant alive in the last quarter | Health outcome sensitivity analyses |
| Year 4 vision bolt-on EQ-5D utility | Regress, pmm | Full model, conditional on participant alive in the last quarter | Health outcome sensitivity analyses |
| *Notes:* Abbreviations: N/A, none applicable. EQ-5D, European quality-of-life five domain. ETDRS, early treatment diabetic retinopathy study. Pmm, predictive mean matching. Anti-VEGF, anti-vascular endothelial growth factor. | | | |

## Cost-utility analysis

The base case within-trial economic evaluation compared the cost-effectiveness of SRT plus anti-VEGF versus anti-VEGF monotherapy from the NHS perspective over a two-year period following an intention-to-treat approach. The incremental cost-effectiveness ratio (ICER), representing the cost per additional quality-adjusted life year (QALY) gained, was calculated as the difference in mean costs divided by the difference in mean QALYs between the two trial groups. The analysis used NICE's cost-effectiveness thresholds of £20,000 and £30,000 per QALY gained to assess whether SRT plus anti-VEGF is cost-effective.^24^

The net monetary benefit (NMB) quantifies the value of a treatment in monetary terms. NMB was calculated as the health benefits (QALYs gained) times monetary value (difference cost-effectiveness threshold) minus the cost difference between trial comparators. A positive incremental NMB indicated that SRT plus anti-VEGF was cost-effective compared to anti-VEGF monotherapy at the specified threshold. Cost-effectiveness acceptability curves (CEACs), which illustrate the probability that a treatment is cost-effective at various willingness-to-pay thresholds, were generated from bootstrap replications. CEACs were calculated by estimating the proportion of bootstraps where the incremental NMB was positive.

## Sensitivity analyses

We conducted the following sensitivity analyses to assess the effect of changing the assumptions or methods on cost-effectiveness:

- Discount rates applied to costs and QALYs were varied between 0% and 5% to assess the impact of societal preferences for present versus future.
- An outlier with high concomitant medication costs in the anti-VEGF monotherapy group was excluded to determine if this affected the results.
- Using vision bolt-on QALYs was explored to better acknowledge the impact of visual impairment on quality of life.
- Participants who died, withdrew, or were lost to follow-up were excluded to test whether the study’s results remained valid across different populations.
- Excluding participants completing the 2-year visit after the pandemic and focusing on participants who had completed two years of follow-ups by 23rd March 2020 to test whether the study’s results remained valid across different populations
- Controlling for recruitment sites was explored to account for potential differences in anti-VEGF dosing practices and drug selection: we used a mixed-effects model, adjusting for sex at birth, baseline age, baseline EQ-5D-5L health utility, days on-trial during the COVID-19 pandemic, and baseline study eye BCVA as fixed effects, while stratifying participant recruitment sites as a random effect.
- Monitoring visits: assuming that participants have monitoring visits only every 3 months after the first year of the trial (with no change in the number of anti-VEGF injections).
- Monitoring visits: including the cost of monthly visits that were cancelled/missed – assuming perfect monitoring visits attendance in years 3 & 4 (until death).
- Monitoring visits: assuming participants had OCT only when they had an injection (treat and extend).
- Adjusting the license fee payable to Zeiss for each NHS treatment of SRT (currently £1,250) within a range of 80% to 160% of the base case.
- In the trial, patients were reimbursed for their transport costs to SRT treatment centres; these costs were excluded from the base case. Sensitivity analysis included participant transport costs for SRT at £206.64 per participant. This figure is based on the £175 received by trial participants to cover the cost of travelling for SRT but was inflated using the Personal Social Services Pay & Prices Index^8^ from the median recruitment year of 2017 to 2021 values.
- Discounts on the price hospitals pay for anti-VEGF drugs prices between 20% to 80%
- Assuming that all anti-VEGF injections were bevacizumab (Avastin) and applying the unit cost of either bevacizumab (Avastin) but not changing the injection frequency.
- Assuming that all anti-VEGF injections were aflibercept (Eylea) and applying the unit cost of aflibercept (Eylea) but not changing the injection frequency.

## Justification for no subgrouping or distributional analysis

None of the planned subgroup analyses on the primary endpoint showed a statistically significant interaction with the primary endpoint at the 5% level.^7^ We therefore did not conduct subgroup analyses other than those testing the robustness of the results to missing data or COVID-19.

No distributional analysis was conducted as no relevant sociodemographic data were collected.

## List of assumptions used within the analysis

### Primary care and concomitant medications—Primary care

- We estimated the cost of primary consultations related to the study eye (or regarding both eyes) based on the responses to the questions: “Did the patient have a community consultation”, who did they see, and what consultation type. The date of the community consultation was used to estimate the time of this visit relative to the participant randomisation.
- If the “no” box was checked for “Did the patient have a community consultation” and no response to the following questions, we consider no resource use of any kind. If the “no” box was checked but responded to the following questions, costs were applied based on the responses to the following answer.
- If the "yes" box was checked for community consultation without any additional selections regarding who they see and what consultation type, the cost of this visit was calculated using the average cost of community consultations where the "yes" box was checked within the first 2 years (£27.6605). This calculation was applied regardless of whether the visit was study eye-related or not, as without further details, the visit may or may not be related to the study eyes.
- We included the cost of eye tests for community optometrists or dispensing opticians that were reported by participants, up to a maximum of one per participant per two-years. This was because all participants on STAR were eligible for at least one NHS-funded community optometrist visit up to year 2 (two if up to year 4). Any subsequent community optometrist visits would be paid out of pocket. If the nurse responded on behalf of a participant for other community consultations and specified a visit of optometrist, opticians, optom, the cost was considered as one community optometrist, applying the 2022 NHS general ophthalmic services fee of £22.14.^9^ The cost of glasses was not included. Diabetic screening tests were costed as general ophthalmic services.
- Consultations reported as “ophthalmologist”, “st thomas”, “low vision clinic-telephone consultations” and those naming a specific opthalmologist were crosschecked with the form documenting hospital eye consultations. If these consultations were not on the same day as a hospital ophthalmology consultation, they were considered as one ophthalmology consultation. In cases where the date was missing and reported after 2 years, it was considered a routine visit for monitoring nAMD.
- Community consultation for “Multiple sclerosis support nurse” and “pre-med for knee” were excluded as they were considered very unlikely to be related to the eyes.
- Consultation with pharmacists, dentists, and vaccinations were excluded as the study intervention was not expected to change the frequency and not all such consultations were funded by the NHS.
- If the “yes” box was checked for a GP visit without specifying the type of visit, the cost of this visit was estimated using the average cost of GP consultation within the first 2 years (£38.16637).
- After 2 years, if a visit was reported with the specification "surgery/clinic" without specifying whether it was a nurse or a GP, the cost was calculated based on the average cost of surgery/clinic visits across GPs, GP nurses, and district nurses between years 3 and 4 (£35.06571).

### Primary care and concomitant medications— Concomitant medications

- The Medical Dictionary for Regulatory Activities (MedDRA, version 17.1, September 2014) organ categories that were specified a priori as having a plausible relationship to the study intervention include: cardiac disorders, eye disorders, neoplasms, and nervous system disorder (including stroke). For each of these body system categories, we first assessed whether there was a statistically significant difference between groups in the mean number of new concomitant medications started during the first two years of the trial (excluding anti-VEGF for eye disease). Following the health economic analysis plan, the cost of concomitant medications in that organ class was included only if this test was statistically significant, to reduce the risk that important differences in medication use between groups were missed. Using a two-tailed t-test, we found p-values of 0.2421 for cardiac disorders, 0.1411 for eye disorders (excluding anti-VEGF), 0.6040 for neoplasms, and 0.0831 for nervous system disorder (including stroke). Using the pre-specified significance level of alpha=0.1, we included the cost of concomitant medications categorized as nervous system disorders (including stroke) throughout the trial.
- Study CRFs reported the name of the medication, body system, date started, ongoing status at study end, and date stopped. These dates were used to estimate the duration of the participant's medication use and the associated cost relative to participant randomisation time. The unit cost was sourced from Prescription Cost Analysis – England – 2021/22,^11^ with the assumption that each prescription lasts 28 days.
- We assumed that participants who were randomised but were not in the concomitant medication dataset had no concomitant medications. The t-test on the numbers of new medications also excluded participants who withdrew or died before year 2 or did not attend the 2-year visit, leaving a total of 241 participants on SRT plus anti-VEGF and 118 on anti-VEGF monotherapy.
- Following the analysis plan, we excluded anti-VEGF drugs reported for use in the body system of eye disorders but retained them for other body systems. This was because drugs like bevacizumab (Avastin) were used to treat specific eye diseases and various cancers.
- If a medication was started on the same day or after it was discontinued, we considered it as a new concomitant medication.
- We used the body system reported for the medication as specified on the CRF.
- In cases where the medication start date was missing or incomplete, if a participant was randomised in 2019 and reports starting a medication within that year without specifying the date or month, we assumed they started the medication before randomisation. Similarly, for participants randomised in May 2018, if the medication was reported to have started in May 2018 without an exact date, we assumed the medication started predate randomisation. If the start date was entirely missing, we assumed that medication was started before randomisation.
- If the day in the medication start/stop date was missing (i.e., no specific day was provided), and the year and month of start/stop were after randomisation, we assumed that the drug was started/stopped on the 15th of that month. If the month in the medication start/stop was missing (i.e., only the year is available) and the year of start/stop was after randomisation, we assumed that the medication was started/stopped on 30th June of that year.
- We assumed any medication that did not end at the study end was ongoing until the participant withdrew, died or reached the planned fourth anniversary of randomisation.
- For nervous system drugs, we calculated the cost of all medications started after randomisation. Medications that participants were taking at randomisation were excluded from the analysis as these are unlikely to be affected by treatment.
- For a given generic name or brand name of the concomitant medications, the unit price was calculated as the weighted average of items prescribed under that chemical substance name. This was because the generic drug was the preferred option and was used more often by the NHS.^25^
- The unit price calculation excluded dosage forms including paediatric, suppositories, oral suspension/liquid/solution/drops, rectal solution tubes, effervescent tablets, and emulsion.
- The scheduled time of the 1-year visit was 336 days and the scheduled time of the 2-year visit was 672 days. The three-year visit was 1008 days after randomisation and the year visit was scheduled to be 1344 (48*28) days after randomisation. Medication costs were estimated up to the scheduled visit time and medication use after that point was excluded.

### Hospital care—consultations and procedures

- We cost hospital consultations related to the study eye (or regarding both eyes) based on the responses to “Did the patient have a Hospital eye consultation”, speciality, procedure(s) or treatment received, and type of consultation. The date of the hospital eye consultation was used to estimate the time of this visit relative to the participant randomisation.
- If the “no” box was checked for “Did the patient have a Hospital eye consultation” and no response to the following questions, we considered no resource use of any kind. If the “no” box was checked but responded to the following questions, costs were applied based on the responses to the following answer.
- To ensure the precision of healthcare resource use measurement related to the study eye, reported hospital visits associated with fellow eyes were omitted.
- We included consultations related to Ophthalmology, A&E, diagnostic imaging, ENT, plastic surgery, trauma, neurology, and orthoptics in the analysis, as they were considered potentially relevant to the study eye. We excluded consultations related to Rheumatology, General Surgery, General Medicine, Endocrinology, Orthopaedics, Urology, Cardiology, and Clinical Haematology, as they were considered unlikely to be related to the eyes.
- Among the specified other procedure(s) or treatment received, no cost was applied to optical coherence tomography, eye drops, best-corrected visual acuity or slit lamp, as these procedures were a part of normal outpatient monitoring visits (counted separately). No cost was applied to ocular pulse amplitude as it was primarily a test for stroke risk and would not be normally done as part of NHS care. No cost was applied to procedures the participant reported as done privately. We applied the cost of one additional medical ophthalmology outpatient service to specified procedures of phasing, low vision assessment, visual field testing and prism (£126).^12^ In cases where multiple procedures occurred on the same day, we assumed that all of them were conducted during one visit.
- The cost of outpatient visits was based on the response to the "type of consultation" question (e.g., first or follow-up visit). For unspecified visits, the cost was calculated as the weighted average of all types of visits.
- The unit cost of procedures was calculated by the weighted average of elective and non-elective for all inpatient procedures, weighted by the number of finished consultant episodes.
- We assumed that the consultation type for Phacoemulsification Cataract Extraction and Lens Implant was day case, irrespective of the response to the type of consultation question. This assumption stemmed from the prevalent practice of UK outpatient clinics performing this procedure in a few alternative settings.^11^
- We applied a weighted average across all consultation types if the reported type was not applicable in HRG: for instance, “retinal repair” reported as emergency consultations was assumed to be the weighted average across all consultation types as HRG code BZ86B were not applicable for emergency care.
- The cost of hospital telephone consultations was estimated as the average of GP and practice nurse phone calls because both services involve similar levels of clinician time and administrative resources. As no specific cost data for hospital telephone consultations were available, the consultations in years three and four were estimated using the average cost of GP and practice nurse phone calls from years one and two (£22.8835).
- If treatment or procedures were completed during an ophthalmology visit, the cost of this visit was calculated through the cost of the treatment or procedures.
- To prevent double counting the injection costs, hospital visits reported with anti-VEGF injections were excluded. This exclusion was justified by the comprehensive documentation of these injections in the Ranibizumab injection log in the first 2 years.
- After 2 years, hospital visits related to ophthalmology for a participant were excluded if they aligned with the dates documented in the Anti-VEGF Injection Administration Log Study eye or Anti-VEGF Injection Administration Log Non-study eye.

### Hospital care— Hospitalisation

- We cost the hospitalisation related to the study eye (or regarding both eyes) based on the responses to “Did the patient have a hospitalisation”, reason for admission/procedure(s) conducted, number of days in hospital and speciality of the ward. The date of the hospital admission was used to estimate the time of this visit relative to the participant randomisation.
- If the “no” box was checked for “Did the patient have a hospitalisation” and no response to the following questions, we assumed no resource use of any kind. If the “no” box was checked but responded to the following questions, costs were applied based on the responses to the following answer.
- If the "yes" box was checked for hospitalisation without any additional selections regarding the reason for admission/procedure(s) conducted, number of days in hospital and speciality of the ward, the cost of this visit was calculated using the average cost of hospitalisation where the "yes" box was checked within the first 2 years (£577.4938). This calculation was applied regardless of whether the visit was study eye-related or not, as without further details, the visit may or may not be related to the study eyes.
- We considered "enhanced cataract surgery" as equivalent to "Phacoemulsification Cataract Extraction and Lens Implant." The cost of these cataract surgeries was calculated using the weighted average of HRG codes BZ34-A, -B, or -C, with weights assigned by the number of finished consultant episodes.^11^ This assumption was based on the understanding that phacoemulsification was the standard pan-UK technique.
- We cost “Non-Surgical Ophthalmology with the length of stay one day or less” as the weighted average of short stay and daycase of with intervention and without intervention (BZ24D, BZ24E, BZ24F and BZ24G).
- We cost “Non-Surgical Ophthalmology with the length of stay 2 days” as the weighted average of short and long stays with intervention and without intervention (BZ24D, BZ24E, BZ24F and BZ24G).
- If a nurse reported on behalf of a participant that they have procedures in “Nasal cautery”, “Elective prostate operation”, “Elective knee operation”, “Cortisone injection in the right thumb”, “cortisone injection”, “Emergency procedure Colostomy ruptured bowel Diverticulitis”, “Overnight stay to AMU due to stomach pain”, “Abdominal pain”, “Nasal fracture due to fall”, “Trauma fractured hip”, “Fractured hip Trauma Surgery performed”, “Atrial flutter”, and “Chest pain”, this visit was excluded as they were considered very unlikely to be related to the eyes.

### EQ-5D Utilities and QALYs

- The scheduled time of the 1-year visit was 336 days and the scheduled time of the 2-year visit was 672 days. The three-year visit was 1008 days after randomisation and the year visit was scheduled to be 1344 (48*28) days after randomisation. We therefore counted 1-year EQ-5D as being at 336 days (not 365 days), 2-year EQ-5D as being at 672 days, three-year EQ-5D as being 1008 days and four-year EQ-5D as being 1344 days. QALYs were therefore calculated over the same number of days to match the scheduled time of visit.

### Year 3/4 –Identifying monitoring regimen for each participant

- nAMD monitoring beyond 2 years was assumed to follow one of three monitoring regimens:
  - Treat-and-extend (T&E): participants have an injection every visit, but the visit duration is modified based on disease activity. Treat and extend regimen will have a maximum interval of 16 weeks.

o    Pro Re Nata (PRN) treatment: the visit interval is fixed at 4 weeks but the injection might or might not occur depending on disease activity.

o   Observation: the disease status is stable and involves no treatment.

- Without referencing the randomisation allocation, each participant was classified into one of the three monitoring regimens based on the clinician's opinion. Participants can switch between regimens during the study period. The following sources were used to support the clinical judgment in participant monitoring regimes in years three and four:

o Based on NICE and drug license posology that were around at the relevant time, aflibercept (Eylea) and faricimab (Vabysmo) are assumed to be monitored as T&E unless there is evidence to the contrary. Ranibizumab (Lucentis) and bevacizumab (Avastin) could be either T&E or PRN. The date of the 2-year visit is used to identify the length of the gap before the first injection date. Alongside the drug received from the study eye, injection dates of the study eye and non-study eyes are used to determine which monitoring regimen each participant is in.

o   If a participant has not withdrawn/died/lost to follow-up in years 3 and 4, but experiences any period of ≥ 16 weeks where no injection was administered, we assume they are subject to an observation monitoring regimen from the last injection up until the point when injections resume, or the participant has reached a follow-up end-point (e.g., died/withdrawn/completed follow up)

- The cost for monitoring AMD with Optical Coherence Tomography (OCT) was estimated by the duration that each participant spent on each regimen from the actual 2-year visit day up to the planned 3-year visit date and from the planned 3-year visit date up to the 4-year visit day.
  - T&E: participants were assumed to have an OCT for each intravitreal injection and we applied the cost of one OCT for every injection given.
  - PRN: The cost of one OCT was applied per 28 days, assuming 0.035714286 (=1/28) OCTs per day on this regimen.
  - Observation: The cost of one OCT was applied every 16 weeks, assuming 0.010951403 (=4/365.25) OCTs per day on this regimen.
- If a participant died in years 3 and 4, we estimated their monitoring costs until the death date.
- If a participant withdrew in years 3 and 4, we continued to estimate participants' monitoring costs if they withdrew consent without further reasons, withdrew to switch to other anti-VEGF drugs, or withdrew to switch to other or NHS care, because under these three cases as they were likely to continue nAMD treatment.
- If a participant received an anti-VEGF injection without specifying the drug, we applied the weighted average drug cost of all injections in the respective year (£747.2006 per dose in year 3 and £768.3653 per dose in year 4).

### Injection and monitoring visit

- Target dates rather than visit dates were used to calculate the number of injections in years 3 and 4. For participants whose 4-year visit date preceded the target date, we assumed the visit was conducted on the target date. For participants with injection dates beyond the 4-year visit, we excluded any that fall after the target date of the 4-year visit.
- After the 2-year visit, the annual number of monitoring visits was estimated by dividing the yearly monitoring cost by the unit cost of each monitoring visit.

### Days on-trial during the COVID-19 pandemic

- To overcome any bias in the impact of COVID-19 restrictions on the possible imbalance in randomisation dates between groups we adjusted for days on trial during the pandemic in regression analyses.
- In years 1-2, we considered days affected by the COVID-19 pandemic from 23/03/2020 to the second anniversary of participant randomisation, which was assumed to be the 672 days after randomisation.
- For years 3-4, we defined the affected days as the period between the participant's second anniversary (672 days after randomisation) and 27/01/2022, on which day England returned to Plan A and stopped the requirement on face coverings and COVID-19 Passes.^26^

### Multiple imputation

- Before the two-year visit, we assumed that if participants missed a visit, the resource usage would be reported on the next visit, given that the questionnaire asked them about primary/hospital consultation and hospitalization since their last attendance at a STAR clinic.
- Before the two-year visit, we treated all the resources used for that participant in that quarter as missing if a participant withdrew at any point during a quarter.
- If a participant withdrew from the study, we assumed they continued taking their medication until the next anniversary of randomisation. This was done to avoid needing to impute missing days on concomitant medication. The concomitant drugs following the year of withdrawal were imputed to take into account cases where a participant may report new concomitant medications after withdrawal. For example, if a participant started taking drug X in year one, and withdrew at 18 months, we assumed they continued drug X until year 2 and imputed the concomitant medications in year 3 and year 4.
- In cases where a participant withdrew from the study before the month 1 visit, the cost of concomitant medications was imputed.
- Following best practice,^21^ we employed mean imputation for missing baseline utility, setting it equal to the mean of non-missing baseline EQ-5D health utility values.
- Considering the low mortality rate (0.7% within the two-year observation), we assumed that anyone who withdrew during the study period remained alive until the four-year follow-up.
- To estimate the quality of life and costs for participants who withdrew, we imputed costs and QALYs conditional on patients being alive. This approach avoided multiple mortality estimates.
- We assumed that participants who withdrew had no hospitalizations related to the study eye after the 2-year visits, as there were no reported hospitalizations related to the study eye among participants who remained in the study.
- For participants who withdrew due to an adverse event, an intercurrent illness that prevents continuing, no longer able to travel to the centre, unable to locate/contact the participant, want to stop treatment altogether, or loss to follow-up, we assumed that there were no further injections or monitoring visits from the withdrawal date onwards. This assumption was because these reasons for withdrawal would generally mean cessation of injections and attendance at NHS clinics. Loss to follow-up was specifically included because extensive efforts are made to contact participants, and if they choose not to be contacted, it suggests the likelihood of disengagement from any eye interventions. The same assumption applied to those unable to attend appointments, as a lack of attendance at one single study visit in 1 year implied non-engagement with other regular visits, especially considering the injection and monitoring cannot be provided through home visits.
- For participants who withdrew consent without further reasons, or withdrew to switch to other anti-VEGF drugs or NHS care, we used multiple imputations to impute data on the numbers of injections and monitoring visits from the last visit onwards. This was because we have no information on participants changed their nAMD treatment approach.
- After the 2-year visit, participants revisit the study site annually. To capture resource use prior to the participant's death, we include the imputed costs of primary visits, outpatient visits, and concomitant medications for the year of death, which are then adjusted based on the proportion of the year the participant lived.

# Supplementary Results

### Table S3. Response levels for vision bolt-on at each time point (available cases)

|  | **Anti-VEGF monotherapy** | | | **SRT plus anti-VEGF** | | | **Difference**  **(SRT plus anti-VEGF minus anti-VEGF monotherapy)** |
| --- | --- | --- | --- | --- | --- | --- | --- |
|  | **No problems (%)** | **Some problems (%)** | **Extreme problems (%)** | **No problems (%)** | **Some problems (%)** | **Extreme problems (%)** | **p-value*** |
| Baseline (n = 401) | 51 (37.5) | 71 (52.21) | 14 (10.29) | 104 (39.25) | 142 (53.58) | 19 (7.17) | 0.56 |
| Week 48 (n = 375) | 45 (36.89) | 68 (55.74) | 9 (7.38) | 109 (43.08) | 130 (51.38) | 14 (5.53) | 0.47 |
| Week 96 (n = 362) | 55 (45.83) | 56 (46.67) | 9 (7.50) | 101 (41.74) | 122 (50.41) | 19 (7.85) | 0.76 |
| Week 144 (n = 342) | 43 (37.72) | 63 (55.26) | 8 (7.02) | 81 (35.53) | 120 (52.63) | 27 (11.84) | 0.38 |
| Week 192 (n = 326) | 31 (29.52) | 61 (59.05) | 12 (11.43) | 76 (34.39) | 117 (52.94) | 28 (12.67) | 0.58 |
| *Notes:* (%) represents the percentage of vision bolt-on response levels to the number of participants in the control group or comparison group at that time point.* Pearson chi-squared test is used to test the difference between trial comparators. Outcomes for the anti-VEGF monotherapy group are from those randomised to sham SRT.^7^ Abbreviations: SRT, stereotactic radiotherapy; VEGF, vascular endothelial growth factor. | | | | | | | |

### Table S4. VFQ subscales at each time point (available cases)

|  | **Sham (anti-VEGF alone)** | **SRT (plus anti-VEGF)** | **Difference (SRT minus sham)*** | | **Cohen's d** |
| --- | --- | --- | --- | --- | --- |
|  | **Mean (SD)** | **Mean (SD)** | **Mean (95% CI)** | **p-value** |  |
| **VFQ: General Vision** | | | | | |
| Baseline (n = 404) | 58.94 (21.81) | 61.28 (19.42) |  |  |  |
| Week 48 (n = 377 ) | 60.12 (20.81) | 63.19 (18.95) | 2.51 (-1.44, 6.46) | 0.21 | 0.16 |
| Week 96 (n = 360) | 61.97 (22.04) | 62.66 (19.91) | 0.90 (-3.43, 5.23) | 0.68 | 0.03 |
| Week 144 (n = 345) | 57.68 (22.83) | 57.58 (21.79) | -1.01 (-5.73, 3.72) | 0.68 | 0.00 |
| Week 192 (n = 330) | 56.84 (22.50) | 53.48 (22.80) | -3.11 (-8.20, 1.97) | 0.23 | -0.15 |
| **VFQ: Ocular Pain** | | | | | |
| Baseline (n = 404) | 88.41 (17.46) | 89.23 (13.96) |  |  |  |
| Week 48 (n = 377 ) | 87.70 (17.23) | 89.90 (14.07) | 1.44 (-1.63, 4.51) | 0.36 | 0.14 |
| Week 96 (n = 360) | 88.76 (15.47) | 89.47 (14.52) | 0.18 (-3.01, 3.37) | 0.91 | 0.05 |
| Week 144 (n = 345) | 86.95 (18.55) | 89.12 (16.03) | 1.01 (-2.57, 4.58) | 0.58 | 0.13 |
| Week 192 (n = 330) | 86.20 (19.21) | 87.78 (16.59) | 1.01 (-2.93, 4.95) | 0.61 | 0.09 |
| **VFQ: Near Vision** | | | | | |
| Baseline (n = 404) | 71.05 (24.91) | 75.03 (21.58) |  |  |  |
| Week 48 (n = 377 ) | 74.66 (24.21) | 75.15 (23.43) | 0.51 (-4.18, 5.19) | 0.83 | 0.02 |
| Week 96 (n = 360) | 72.62 (25.19) | 73.86 (24.28) | 1.14 (-3.89, 6.17) | 0.66 | 0.05 |
| Week 144 (n = 345) | 68.93 (27.15) | 70.69 (25.58) | 0.55 (-4.83, 5.92) | 0.84 | 0.07 |
| Week 192 (n = 330) | 70.01 (26.25) | 66.74 (26.99) | -3.01 (-8.83, 2.81) | 0.31 | -0.12 |
| **VFQ: Distance Vision** | | | | | |
| Baseline (n = 404) | 76.64 (22.25) | 81.78 (20.29) |  |  |  |
| Week 48 (n = 377 ) | 78.24 (22.49) | 81.26 (20.85) | 2.93 (-1.18, 7.04) | 0.16 | 0.14 |
| Week 96 (n = 360) | 76.54 (24.37) | 80.31 (22.33) | 3.19 (-1.56, 7.95) | 0.19 | 0.16 |
| Week 144 (n = 345) | 75.30 (26.33) | 77.07 (24.45) | 0.25 (-4.95, 5.46) | 0.92 | 0.07 |
| Week 192 (n = 330) | 73.82 (27.54) | 74.74 (25.66) | 0.76 (-4.81, 6.33) | 0.79 | 0.03 |
| **VFQ: Social Functioning** | | | | | |
| Baseline (n = 404) | 88.32 (19.31) | 91.25 (17.61) |  |  |  |
| Week 48 (n = 377 ) | 88.43 (18.55) | 90.70 (17.93) | 2.34 (-1.30, 5.98) | 0.21 | 0.13 |
| Week 96 (n = 360) | 85.92 (23.05) | 89.42 (19.39) | 3.53 (-0.67, 7.72) | 0.10 | 0.17 |
| Week 144 (n = 345) | 85.53 (23.32) | 87.23 (21.93) | 0.81 (-4.02, 5.64) | 0.74 | 0.08 |
| Week 192 (n = 330) | 85.14 (23.79) | 86.15 (21.54) | 1.07 (-3.87, 6.01) | 0.67 | 0.05 |
| **VFQ: Mental Health** | | | | | |
| Baseline (n = 404) | 73.08 (26.71) | 76.78 (23.28) |  |  |  |
| Week 48 (n = 377 ) | 74.57 (26.64) | 78.95 (24.13) | 3.86 (-1.13, 8.85) | 0.13 | 0.17 |
| Week 96 (n = 360) | 75.21 (25.74) | 77.37 (24.68) | 2.12 (-2.98, 7.23) | 0.41 | 0.09 |
| Week 144 (n = 345) | 71.11 (28.20) | 74.19 (25.97) | 1.48 (-4.12, 7.08) | 0.60 | 0.12 |
| Week 192 (n = 330) | 71.09 (28.35) | 71.64 (27.11) | 0.04 (-5.86, 5.94) | 0.99 | 0.02 |
| **VFQ: Role Difficulties** | | | | | |
| Baseline (n = 404) | 77.10 (26.97) | 78.90 (24.25) |  |  |  |
| Week 48 (n = 377 ) | 76.13 (28.30) | 79.53 (25.55) | 3.03 (-2.33, 8.39) | 0.27 | 0.13 |
| Week 96 (n = 360) | 74.79 (27.66) | 76.93 (28.33) | 1.92 (-3.79, 7.63) | 0.51 | 0.08 |
| Week 144 (n = 345) | 70.35 (29.51) | 73.58 (29.42) | 1.14 (-4.94, 7.23) | 0.71 | 0.11 |
| Week 192 (n = 330) | 70.0 (30.94) | 71.65 (29.33) | 1.28 (-5.31, 7.87) | 0.70 | 0.06 |
| **VFQ: Dependency** | | | | | |
| Baseline (n = 404) | 86.34 (24.08) | 90.76 (20.42) |  |  |  |
| Week 48 (n = 377 ) | 86.61 (25.30) | 89.02 (22.24) | 1.76 (-2.83, 6.35) | 0.45 | 0.10 |
| Week 96 (n = 360) | 84.10 (26.04) | 88.13 (22.66) | 3.97 (-0.97, 8.90) | 0.11 | 0.17 |
| Week 144 (n = 345) | 81.64 (28.07) | 84.29 (26.10) | 1.16 (-4.46, 6.77) | 0.69 | 0.10 |
| Week 192 (n = 330) | 79.60 (30.36) | 81.22 (27.79) | 1.15 (-5.07, 7.37) | 0.72 | 0.06 |
| **VFQ: Driving** | | | | | |
| Baseline (n = 404) | 68.50 (35.11) | 73.39 (29.24) |  |  |  |
| Week 48 (n = 377 ) | 64.77 (36.66) | 70.88 (33.74) | 7.72 (-0.33, 15.77) | 0.06 | 0.18 |
| Week 96 (n = 360) | 56.80 (40.16) | 66.01 (35.88) | 8.98 (-0.05, 18.01) | 0.05 | 0.25 |
| Week 144 (n = 345) | 57.18 (40.77) | 59.90 (38.45) | 1.85 (-8.0, 11.69) | 0.71 | 0.07 |
| Week 192 (n = 330) | 51.75 (42.24) | 56.51 (39.0) | 7.16 (-3.42, 17.74) | 0.18 | 0.12 |
| **VFQ: Colour Vision** | | | | | |
| Baseline (n = 404) | 92.10 (18.68) | 94.45 (14.40) |  |  |  |
| Week 48 (n = 377 ) | 94.67 (13.72) | 93.87 (15.33) | -0.87 (-4.0, 2.26) | 0.59 | -0.05 |
| Week 96 (n = 360) | 92.37 (17.76) | 93.49 (15.90) | 1.32 (-2.21, 4.85) | 0.46 | 0.07 |
| Week 144 (n = 345) | 91.67 (17.18) | 93.37 (15.39) | 1.34 (-2.21, 4.89) | 0.46 | 0.11 |
| Week 192 (n = 330) | 92.38 (17.38) | 90.95 (19.29) | -1.56 (-5.87, 2.76) | 0.48 | -0.08 |
| **VFQ: Peripheral Vision** | | | | | |
| Baseline (n = 404) | 80.51 (24.85) | 87.36 (20.43) |  |  |  |
| Week 48 (n = 377 ) | 83.54 (23.48) | 86.01 (21.64) | 1.71 (-2.77, 6.19) | 0.45 | 0.11 |
| Week 96 (n = 360) | 80.04 (26.16) | 85.17 (21.57) | 4.30 (-0.38, 8.99) | 0.07 | 0.22 |
| Week 144 (n = 345) | 78.86 (25.38) | 81.86 (24.21) | 1.65 (-3.71, 7.01) | 0.54 | 0.12 |
| Week 192 (n = 330) | 81.43 (25.01) | 82.61 (23.32) | 0.15 (-5.22, 5.53) | 0.96 | 0.05 |
| *Notes:* Differences and p-values derived from the ordinary least squares regression model adjusted for sex at birth, baseline age, baseline EQ-5D health utility, days on-trial affected by COVID-19 pandemic, and study eye best-corrected visual acuity. . **^b^** Calculated using available cases. An effect size based on Cohen’s d is typically interpreted as small when between 0.2 and 0.5, moderate when between 0.5 and 0.8, and large when greater than 0.8 ^27^. Abbreviations: CI, confidence interval; SD, standard deviation; SRT, stereotactic radiotherapy; VEGF, vascular endothelial growth factor; VFQ, Visual Function Questionnaire. | | | | |  |

### Table S5. QALY with and without vision bolt-on at each time point

|  | **Anti-VEGF monotherapy** | **SRT plus anti-VEGF** | **Difference**  **(SRT plus anti-VEGF minus anti-VEGF monotherapy)*** | |
| --- | --- | --- | --- | --- |
|  | **Mean (SD)** | **Mean (SD)** | **Mean (95% CI)** | **p-value** |
| **QALYs†** | | | | |
| Year 1 | 0.82 (0.17) | 0.83 (0.17) | 0.00 (-0.01, 0.02) | 0.83 |
| Year 2 | 0.79 (0.20) | 0.79 (0.21) | 0.00 (-0.03, 0.03) | 0.86 |
| Year 3 | 0.75 (0.25) | 0.76 (0.26) | 0.00 (-0.04, 0.05) | 0.89 |
| Year 4 | 0.73 (0.26) | 0.74 (0.28) | 0.01 (-0.04, 0.05) | 0.81 |
| **QALYs with vision bolt-on†** | | | | |
| Year 1 | 0.79 (0.19) | 0.80 (0.18) | 0.01 (-0.01, 0.02) | 0.48 |
| Year 2 | 0.77 (0.22) | 0.77 (0.22) | 0.00 (-0.03, 0.03) | 0.96 |
| Year 3 | 0.72 (0.26) | 0.72 (0.28) | 0.00 (-0.05, 0.05) | 0.98 |
| Year 4 | 0.69 (0.27) | 0.70 (0.29) | 0.01 (-0.04, 0.05) | 0.83 |
| *Notes:* *Differences and p-values derived from the ordinary least square model adjusted for sex at birth, days on-trial affected by COVID-19 pandemic, baseline age, utility, and study eye BCVA. †QALYs were not discounted for time preference. Outcomes for the anti-VEGF monotherapy group were from those randomised to sham SRT.^7^ Abbreviations: CI, confidence interval; QALY, quality-adjusted life year; SD, standard deviation; SRT, stereotactic radiotherapy; VEGF, vascular endothelial growth factor. EQ-5D utility scores with the bolt-on are slightly lower than the those without the bolt on as the bolt-on utilities were estimated by subtracting an additional coefficient using published tariff adjustments (18). | | | | |

Table S6: Vision and health-related quality of life across vision bolt-on levels

| Measure | **Total (n=1806)** | **EQ-5D-3L bolt-on response level**  **Vision (using glasses or contact lenses if needed)** | | |
| --- | --- | --- | --- | --- |
|  |  | **No problems (n=696)** | **Some problems (n=951)** | **Extreme problems (n=159)** |
|  | **Mean (SD)** | **Mean (SD)** | **Mean (SD)** | **Mean (SD)** |
| BCVA: Better-seeing Eye | 76.44 (11.70) | 81.20 (6.77) | 75.71 (10.53) *** | 60.04 (17.89) *** |
| BCVA: Worse-seeing Eye | 55.53 (23.55) | 64.95 (18.88) | 52.61 (23.03) *** | 31.78 (23.75) *** |
| EQ5D: Utility scores valued from Hernandez Alva crosswalk | 0.81 (0.21) | 0.90 (0.16) | 0.78 (0.20) *** | 0.60 (0.28) *** |
| EQ5D: Utility scores using England 5L value set | 0.87 (0.18) | 0.93 (0.13) | 0.85 (0.17) *** | 0.68 (0.25) *** |
| EQ5D: Visual Analogue Scale | 80.93 (16.39) | 87.11 (12.74) | 79.06 (15.72) *** | 65.34 (20.84) *** |
| VFQ: General Vision | 59.60 (21.20) | 73.63 (13.43) | 55.15 (17.41) *** | 25.47 (18.76) *** |
| VFQ: Ocular Pain | 88.65 (15.89) | 93.53 (11.09) | 87.16 (15.75) *** | 75.94 (24.14) *** |
| VFQ: Near Vision | 72.16 (24.82) | 89.22 (13.56) | 66.32 (21.32) *** | 33.23 (21.47) *** |
| VFQ: Distance Vision | 78.20 (23.40) | 91.54 (12.15) | 74.84 (20.91) *** | 39.82 (25.49) *** |
| VFQ: Social Functioning | 88.31 (20.36) | 97.51 (8.61) | 87.02 (18.60) *** | 55.70 (29.78) *** |
| VFQ: Mental Health | 74.98 (25.77) | 90.12 (13.95) | 70.90 (23.51) *** | 33.20 (23.79) *** |
| VFQ: Role Difficulties | 75.51 (27.86) | 91.20 (16.64) | 70.94 (25.15) *** | 34.18 (30.53) *** |
| VFQ: Dependency | 85.91 (24.97) | 97.06 (11.29) | 84.39 (23.81) *** | 45.83 (31.57) *** |
| VFQ: Driving | 64.03 (36.91) | 80.29 (25.89) | 58.32 (37.17) *** | 12.03 (26.39) *** |
| VFQ: Colour Vision | 93.08 (16.39) | 98.22 (7.10) | 92.96 (15.34) *** | 71.63 (28.97) *** |
| VFQ: Peripheral Vision | 83.47 (23.24) | 94.44 (13.40) | 80.80 (21.75) *** | 52.07 (30.35) *** |
| VFQ: Composite Score | 78.84 (18.97) | 90.81 (8.95) | 75.81 (15.76) *** | 44.69 (20.01) *** |
| *Notes:* Available-case means of best-corrected visual acuity (letter scores), EQ-5D utility scores, and VFQ-25 subscale and composite scores were compared across levels of the vision bolt-on responses, using the pooled panel data. A higher value indicates better vision and health-related quality of life for all measures. The better-seeing eye was defined as the eye with the higher visual acuity letter score between the two eyes for each participant at the time of measurement; the worse-seeing eye was the one with the lower score. ***p < 0.01. P-values were derived from the mixed-effects linear regression model adjusted for sex at birth, baseline age, and days on-trial affected by the COVID-19 pandemic, allowing a random intercept for each participant. Abbreviations: BCVA, best corrected visual acuity; SD, standard deviation; VFQ, visual function questionnaire. | | | | |

Table S7: EQ-5D responses across vision bolt-on levels

|  | **Vision (using glasses or contact lenses if needed)** | | |
| --- | --- | --- | --- |
|  | **No problems** | **Some problems** | **Extreme problems** |
|  | Frequency (%) | Frequency (%) | Frequency (%) |
| **Mobility** | | | |
| No problems | 553 (79.45) | 507 (53.31) *** | 46 (28.93) *** |
| Slight problems | 79 (11.35) | 212 (22.29) *** | 29 (18.24) *** |
| Moderate problems | 43 (6.18) | 179 (18.82) *** | 56 (35.22) *** |
| Severe problems | 18 (2.59) | 48 (5.05) *** | 27 (16.98) *** |
| Extreme problems | 3 (0.43) | 5 (0.53) *** | 1 (0.63) *** |
| **Self-Care** | | | |
| No problems | 667 (95.83) | 832 (87.76) *** | 107 (67.30) *** |
| Slight problems | 16 (2.30) | 80 (8.44) *** | 27 (16.98) *** |
| Moderate problems | 8 (1.15) | 29 (3.06) *** | 20 (12.58) *** |
| Severe problems | 3 (0.43) | 5 (0.53) *** | 5 (3.14) *** |
| Unable to | 2 (0.29) | 2 (0.21) * | 0 (0.0) * |
| **Usual Activities *(e.g. work, study, housework, family or leisure activities)*** | | | |
| No problems | 594 (85.71) | 502 (52.84) *** | 34 (21.38) *** |
| Slight problems | 62 (8.95) | 264 (27.79) *** | 35 (22.01) *** |
| Moderate problems | 30 (4.33) | 150 (15.79) *** | 60 (37.74) *** |
| Severe problems | 3 (0.43) | 21 (2.21) *** | 20 (12.58) *** |
| Unable to | 4 (0.58) | 13 (1.37) *** | 10 (6.29) *** |
| **Pain/Discomfort** | | | |
| No pain or discomfort | 499 (71.80) | 436 (45.85) *** | 52 (32.70) *** |
| Slight pain or discomfort | 115 (16.55) | 265 (27.87) *** | 35 (22.01) *** |
| Moderate pain or discomfort | 66 (9.50) | 195 (20.50) *** | 43 (27.04) *** |
| Severe pain or discomfort | 13 (1.87) | 47 (4.94) *** | 27 (16.98) *** |
| Extreme pain or discomfort | 2 (0.29) | 8 (0.84) *** | 2 (1.26) *** |
| **Anxiety/Depression** | | | |
| Not anxious or depressed | 600 (86.46) | 695 (73.16) *** | 71 (44.65) *** |
| Slightly anxious or depressed | 83 (11.96) | 177 (18.63) *** | 41 (25.79) *** |
| Moderately anxious or depressed | 8 (1.15) | 73 (7.68) *** | 35 (22.01) *** |
| Severely anxious or depressed | 1 (0.14) | 2 (0.21) *** | 12 (7.55) *** |
| Extremely anxious or depressed | 2 (0.29) | 3 (0.32) ** | 0 (0.0) ** |
| *Notes:* Available-case means of responses on the five EQ-5D domains were compared across levels of the vision bolt-on responses using pooled panel data. p < 0.1, *p < 0.05, **p < 0.01. Statistical significance was assessed using mixed-effects multinomial logistic regression, with the EQ-5D 5-level response as the outcome and the vision bolt-on category (No/Some/Extreme) as the main predictor. Models were adjusted for sex at birth, baseline age, and the number of on-trial days affected by the COVID-19 pandemic, and included a participant-level random intercept to account for repeated measurements. Model-based effect sizes are summarised as average marginal effects (percentage-point changes in the probability of each response level); see Table 1. | | | |

### Table S8. Coefficients of vision bolt-on on EQ-5D domains

|  | **Vision (using glasses or contact lenses if needed)** | | | | | | | |
| --- | --- | --- | --- | --- | --- | --- | --- | --- |
|  | **Some problems** | | | | **Extreme problems** | | | |
|  | dy/dx | SE | z | P value | dy/dx | SE | z | P value |
| **Mobility** | | | | | | | | |
| No problems | -0.12 | 0.02 | -6.13 | p < 0.01 | -0.29 | 0.03 | -9.18 | p < 0.01 |
| Slight problems | 0.03 | 0.01 | 5.29 | p < 0.01 | 0.08 | 0.01 | 6.90 | p < 0.01 |
| Moderate problems | 0.05 | 0.01 | 5.74 | p < 0.01 | 0.13 | 0.02 | 7.90 | p < 0.01 |
| Severe problems | 0.03 | 0.01 | 5.29 | p < 0.01 | 0.07 | 0.01 | 7.02 | p < 0.01 |
| Extreme problems | 0.00 | 0.00 | 2.90 | p < 0.01 | 0.01 | 0.00 | 3.11 | p < 0.01 |
| **Self-Care** | | | | | | | | |
| No problems | -0.06 | 0.02 | -3.51 | p < 0.01 | -0.14 | 0.02 | -6.29 | p < 0.01 |
| Slight problems | 0.03 | 0.01 | 3.46 | p < 0.01 | 0.08 | 0.01 | 5.83 | p < 0.01 |
| Moderate problems | 0.02 | 0.01 | 3.16 | p < 0.01 | 0.04 | 0.01 | 4.90 | p < 0.01 |
| Severe problems | 0.01 | 0.00 | 2.60 | 0.01 | 0.01 | 0.00 | 3.29 | p < 0.01 |
| Unable to | 0.00 | 0.00 | 1.78 | 0.08 | 0.01 | 0.00 | 1.96 | p < 0.01 |
| **Usual Activities *(e.g. work, study, housework, family or leisure activities)*** | | | | | | | | |
| No problems | -0.21 | 0.02 | -9.37 | p < 0.01 | -0.44 | 0.03 | -12.82 | p < 0.01 |
| Slight problems | 0.08 | 0.01 | 8.21 | p < 0.01 | 0.16 | 0.02 | 9.62 | p < 0.01 |
| Moderate problems | 0.10 | 0.01 | 8.35 | p < 0.01 | 0.20 | 0.02 | 10.81 | p < 0.01 |
| Severe problems | 0.02 | 0.00 | 5.41 | p < 0.01 | 0.05 | 0.01 | 6.07 | p < 0.01 |
| Unable to | 0.02 | 0.00 | 4.46 | p < 0.01 | 0.03 | 0.01 | 4.75 | p < 0.01 |
| **Pain/Discomfort** | | | | | | | | |
| No pain or discomfort | -0.15 | 0.02 | -7.45 | p < 0.01 | -0.28 | 0.03 | -7.96 | p < 0.01 |
| Slight pain or discomfort | 0.04 | 0.01 | 6.05 | p < 0.01 | 0.07 | 0.01 | 6.27 | p < 0.01 |
| Moderate pain or discomfort | 0.07 | 0.01 | 6.88 | p < 0.01 | 0.13 | 0.02 | 7.26 | p < 0.01 |
| Severe pain or discomfort | 0.03 | 0.01 | 5.81 | p < 0.01 | 0.06 | 0.01 | 6.10 | p < 0.01 |
| Extreme pain or discomfort | 0.01 | 0.00 | 3.11 | p < 0.01 | 0.01 | 0.00 | 3.15 | p < 0.01 |
| **Anxiety/Depression** | | | | | | | | |
| Not anxious or depressed | -0.11 | 0.02 | -5.58 | p < 0.01 | -0.28 | 0.03 | -8.72 | p < 0.01 |
| Slightly anxious or depressed | 0.07 | 0.01 | 5.38 | p < 0.01 | 0.16 | 0.02 | 7.81 | p < 0.01 |
| Moderately anxious or depressed | 0.04 | 0.01 | 5.04 | p < 0.01 | 0.09 | 0.01 | 7.18 | p < 0.01 |
| Severely anxious or depressed | 0.01 | 0.00 | 3.14 | p < 0.01 | 0.02 | 0.00 | 3.53 | p < 0.01 |
| Extremely anxious or depressed | 0.00 | 0.00 | 2.11 | 0.04 | 0.01 | 0.00 | 2.21 | 0.03 |

*Notes:* Results were derived from mixed-effects logistic regression models adjusted for sex at birth, baseline age, and days on trial affected by COVID-19 pandemic. Abbreviations: dy/dx, marginal effect; EQ-5D, European quality-of-life five-dimension questionnaire; SE, standard error; z, z-statistic; VFQ, Visual Function Questionnaire.

### Figure S1. Comparison of the number of anti-VEGF injections over time

*Footnotes:* Results were analysed on an intention-to-treat principle with missing data imputed using multiple imputations and including all patients as randomised; values therefore differ slightly from those reported previously. Outcomes for the anti-VEGF monotherapy group were from those randomised to sham SRT 7. The 95% confidence intervals (dotted lines) were estimated using bootstrapping adjusted for sex at birth, baseline age, days on trial during the COVID-19 pandemic, baseline EQ-5D health utility, and baseline study eye best-corrected visual acuity. Mean values in each group are presented for the average participant, assumed to be a woman aged 78 years, who had zero days affected by the COVID-19 pandemic with best-corrected visual acuity of 69 letters and EQ-5D health utility of 0.84. Abbreviations: SRT, stereotactic radiotherapy; VEGF, vascular endothelial growth factor

### Table S9. Number of participants receiving each anti-VEGF drug in years 3 and 4 (available cases)

|  | **Anti-VEGF monotherapy**  **(n = 137 )** | **SRT plus anti-VEGF**  **(n= 251)** | **Total**  **(n=372 )** |
| --- | --- | --- | --- |
| Ranibizumab | 86 | 175 | 261 |
| Bevacizumab | 0 | 1 | 1 |
| Aflibercept | 8 | 23 | 31 |
| Faricimab | 0 | 1 | 2 |
| *Notes:* This table was based on the anti-VEGF injection log for the study eye reported by hospital records. Available cases excluded participants who died (n = 10) or withdrew (n = 29) by year two. Participants could have multiple drugs and switch between drugs in years three and four. Abbreviations: VEGF, vascular endothelial growth factor | | | |

### Table S10. Comparison of resource use quantities in the anti-VEGF monotherapy and SRT plus anti-VEGF groups

|  | **Anti-VEGF monotherapy (n=137)** | **SRT plus anti-VEGF (n=274)** | **Difference**  **(SRT plus anti-VEGF minus anti-VEGF monotherapy)** |
| --- | --- | --- | --- |
| **Years 1-2** | | | |
| Ranibizumab injections: Mean (95% CI) | 13.316 (12.235, 14.438) | 10.927 (9.941, 11.902) | -2.389 (-3.607, -1.162)* |
| Monitoring consultations: Mean (95% CI) | 22.413 (21.756, 23.023) | 22.543 (22.029, 23.012) | 0.130 (-0.450, 0.728) |
| Primary care or concomitant medication: % (95% CI) | 0.360 (0.314, 0.409) | 0.314 (0.209, 0.425) | -0.046 (-0.144, 0.058) |
| Hospital care: % (95% CI) | 0.295 (0.251, 0.341) | 0.303 (0.201, 0.411) | 0.009 (-0.085, 0.106) |
| **Years 3-4** | | | |
| Anti-VEGF injections: Mean (95% CI) | 8.004 (6.558, 9.457) | 7.976 (6.725, 9.207) | -0.028 (-1.346, 1.314) |
| Monitoring consultations: Mean (95% CI) | 10.745 (9.491, 11.992) | 10.532 (9.422, 11.616) | -0.213 (-1.345, 0.897) |
| Primary care or concomitant medication: % (95% CI) | 0.153 (0.117, 0.192) | 0.152 (0.070, 0.248) | -0.001 (-0.078, 0.087) |
| Hospital care: % (95% CI) | 0.127 (0.092, 0.163) | 0.123 (0.047, 0.215) | -0.004 (-0.075, 0.078) |
| *Note:* Results were analysed on an intention-to-treat principle with missing data imputed from multivariate imputation, conditional on participants being alive. (%) represents the proportion of participants with resource use; * represents between-group differences based on two-sided bootstrap p-values (p < 0.05). CIs are estimated using bootstrapping adjusted for treatment allocation, sex at birth, days on-trial affected by COVID-19 pandemic, baseline age, baseline EQ-5D health utility, and baseline study eye BCVA. Mean values in each treatment group are presented for the average participant, assumed to be women aged 78 years, who had zero days affected by the COVID pandemic with BCVA of 69 letters and EQ-5D-5L utility of 0.84. Outcomes for the anti-VEGF monotherapy group are from those randomised to sham SRT.^7^ Abbreviations: CI, confidence interval; SRT, stereotactic radiotherapy; VEGF, vascular endothelial growth factor. | | | |

Figure S2. The probability of SRT plus anti-VEGF being cost-effective compared with anti-VEGF alone at an NHS cost-effectiveness threshold of £20 000 per QALY

*Footnotes:* The base case analysis compared the cost-effectiveness of SRT plus anti-VEGF against anti-VEGF alone over a two- and four-year time horizon at an NHS cost-effectiveness threshold of £20 000 per QALY. Discount rates applied to costs and QALYs were varied between 0% and 5% to assess the impact of societal preferences for present versus future. An outlier with high concomitant medication costs in the anti-VEGF alone group was excluded to determine if this affected the results. Using vision bolt-on QALYs was explored to better acknowledge the impact of visual impairment on quality of life. Participants who died, withdrew, were lost to follow-up, or were influenced by the pandemic were excluded to test whether the study’s results remained valid across different populations. Varying the frequency of monitoring visits was conducted to explore the validity of results under different clinical conditions and capacities. Controlling for recruitment sites was explored to account for potential differences in anti-VEGF dosing practices and drug selection. The SRT license fee was varied between 80% and 160% to explore how changes in licensing costs might affect cost-effectiveness results. In the trial, patients were reimbursed for their transport costs to SRT treatment centres; a sensitivity analysis included these costs to assess whether capturing the full economic burden changed the conclusions. Discounts on the price hospitals pay for anti-VEGF drugs prices between 20% to 80% were explored, and alternative drugs were considered to understand the influence of future price changes and different drug choices on results. Table S11 presents the complete results, including the total cost and QALYs, as well as the differences in cost and QALYs for the year 1-2 and year 1-4 periods. Abbreviations: NHS, national health service; OCT, optical coherence tomography; QALY, quality-adjusted life year; SRT, stereotactic radiotherapy; VEGF, vascular endothelial growth factor.

### Figure S3. Cost-effectiveness acceptability curves for two and four years

*Notes:* Cost-effectiveness acceptability curves showing the probability of base-case and each sensitivity analysis present the best value for money at different thresholds that the NHS may be willing or able to pay (save) to gain (loss) one QALY. The base case analysis compared the cost-effectiveness of SRT plus anti-VEGF against anti-VEGF monotherapy over a two- and four-year time horizon. Discount rates applied to costs and QALYs were varied between 0% and 5% to assess the impact of societal preferences for present versus future. An outlier with high concomitant medication costs in the anti-VEGF monotherapy group was excluded to determine if this affected the results. Using vision bolt-on QALYs was explored to better acknowledge the impact of visual impairment on quality of life. Participants who died, withdrew, were lost to follow-up, or were influenced by pandemic were excluded to test whether the study’s results remained valid across different populations. Varying the frequency of monitoring visits was conducted to explore the validity of results under different clinical conditions and capacities. Controlling for recruitment sites was explored to account for potential differences in anti-VEGF dosing practices and drug selection. The SRT license fee was varied between 80% and 160% to explore how changes in licensing costs might affect cost-effectiveness results. In the trial, patients were reimbursed for their transport costs to SRT treatment centres; these costs were excluded from the base case but a sensitivity analysis included these costs to assess whether capturing the full economic burden changed the conclusions. Discounts on the price hospitals pay for anti-VEGF drugs prices between 20% to 80% were explored, and alternative drugs were considered to understand the influence of future price changes and different drug choices on results. Abbreviations: OCT, optical coherence tomography; QALY, quality-adjusted life year; SRT, stereotactic radiotherapy; VEGF, vascular endothelial growth factor.

### Table S11. Results of sensitivity analysis and subgroup analyses for SRT plus anti-VEGF versus anti-VEGF monotherapy

| **Scenarios** | **Variations** | **SRT plus anti-VEGF (n=274)** | | **Anti-VEGF monotherapy (n=137)** | | **Difference (SRT plus anti-VEGF minus anti-VEGF monotherapy)** | | **Cost/QALY (£)** | **Probability that SRT is** | |
| --- | --- | --- | --- | --- | --- | --- | --- | --- | --- | --- |
|  |  | **Total cost (£)** | **Total QALYs** | **Total cost (£)** | **Total QALYs** | **Total cost (£)** | **Total QALYs** |  | **Cost-effective†** | **Less costly** |
| **Week 0-96** | | | | | | | | | | |
| Base case | | 13,261 (358) | 1.57 (0.02) | 13,725 (411) | 1.57 (0.02) | -464 (-1,344, 420) | 0.00 (-0.04, 0.04) | 417,310 SW | 78% | 85% |
| Discount rate | 0% | 13,435 (365) | 1.60 (0.02) | 13,930 (419) | 1.60 (0.02) | -495 (-1,391, 402) | 0.00 (-0.04, 0.04) | 409,759 SW | 79% | 87% |
|  | 5% | 13,190 (355) | 1.56 (0.02) | 13,641 (408) | 1.56 (0.02) | -451 (-1,323, 426) | 0.00 (-0.04, 0.04) | 420,773 SW | 77% | 85% |
| Excluding concomitant medication costs | | 13,256 (358) | 1.57 (0.02) | 13,710 (409) | 1.57 (0.02) | -454 (-1,328, 429) | 0.00 (-0.04, 0.04) | 408,195 SW | 77% | 85% |
| Vision Bolt-on QALYs | | 13,261 (358) | 1.51 (0.02) | 13,725 (411) | 1.51 (0.02) | -464 (-1,344, 420) | 0.01 (-0.04, 0.05) | Dominant | 83% | 85% |
| Excluding participants who died by 4 years (n=387) | | 13,406 (371) | 1.59 (0.02) | 13,749 (425) | 1.57 (0.02) | -342 (-1,253, 559) | 0.02 (-0.02, 0.06) | Dominant | 90% | 78% |
| Excluding participants who died or withdrew by 4 years (n=335) | | 13,336 (403) | 1.60 (0.02) | 13,763 (441) | 1.59 (0.02) | -426 (-1,412, 551) | 0.01 (-0.03, 0.05) | Dominant | 85% | 81% |
| Primary analysis population: Excluding participants who died, withdrew or were lost to follow-up by 4 years (n=328) | | 13,373 (404) | 1.60 (0.02) | 13,833 (443) | 1.59 (0.02) | -460 (-1,446, 507) | 0.01 (-0.03, 0.05) | Dominant | 83% | 82% |
| Excluding participants completing 2-years visit after pandemic (n=252) | | 13,471 (429) | 1.57 (0.02) | 13,880 (504) | 1.58 (0.02) | -409 (-1,522, 702) | -0.01 (-0.06, 0.03) | 29,113 SW | 57% | 77% |
| Controlling for recruiting sites | | 13,404 (401) | 1.57 (0.02) | 13,953 (429) | 1.57 (0.02) | -549 (-1,401, 223) | 0.00 (-0.04, 0.04) | 493,706 SW | 85% | 93% |
| Monitoring visit | OCT every 3 months after year 1 | 12,189 (352) | 1.57 (0.02) | 12,675 (400) | 1.57 (0.02) | -485 (-1,352, 385) | 0.00 (-0.04, 0.04) | 436,755 SW | 79% | 87% |
|  | Assuming perfect monitoring visit attendance | 13,261 (358) | 1.57 (0.02) | 13,725 (411) | 1.57 (0.02) | -464 (-1,344, 420) | 0.00 (-0.04, 0.04) | 417,310 SW | 78% | 85% |
|  | OCT only when participants have injections | 11,447 (427) | 1.57 (0.02) | 12,303 (484) | 1.57 (0.02) | -857 (-1,907, 198) | 0.00 (-0.04, 0.04) | 770,932 SW | 90% | 95% |
| SRT license fee | 80% | 13,041 (358) | 1.57 (0.02) | 13,725 (411) | 1.57 (0.02) | -683 (-1,561, 199) | 0.00 (-0.04, 0.04) | 614,722 SW | 88% | 94% |
|  | 120% | 13,480 (358) | 1.57 (0.02) | 13,725 (411) | 1.57 (0.02) | -244 (-1,125, 641) | 0.00 (-0.04, 0.04) | 219,899 SW | 65% | 71% |
|  | 140% | 13,700 (358) | 1.57 (0.02) | 13,725 (411) | 1.57 (0.02) | -25 (-908, 863) | 0.00 (-0.04, 0.04) | 22,487 SW | 50% | 53% |
|  | 160% | 13,919 (358) | 1.57 (0.02) | 13,725 (411) | 1.57 (0.02) | 194 (-691, 1,084) | 0.00 (-0.04, 0.04) | Dominated | 35% | 33% |
|  | Including SRT transport costs at £207/patient | 13,467 (358) | 1.57 (0.02) | 13,725 (411) | 1.57 (0.02) | -257 (-1,137, 626) | 0.00 (-0.04, 0.04) | 231,383 SW | 66% | 72% |
| Anti-VEGF injections | 20% discount on ranibizumab | 12,065 (305) | 1.57 (0.02) | 12,270 (350) | 1.57 (0.02) | -206 (-960, 546) | 0.00 (-0.04, 0.04) | 185,173 SW | 63% | 71% |
|  | 30% discount on ranibizumab | 11,471 (278) | 1.57 (0.02) | 11,548 (321) | 1.57 (0.02) | -77 (-769, 608) | 0.00 (-0.04, 0.04) | 69,005 SW | 54% | 59% |
|  | 50% discount on ranibizumab | 10,284 (226) | 1.57 (0.02) | 10,103 (262) | 1.57 (0.02) | 182 (-377, 736) | 0.00 (-0.04, 0.04) | Dominated | 33% | 26% |
|  | 80% discount on ranibizumab | 8,504 (149) | 1.57 (0.02) | 7,935 (176) | 1.57 (0.02) | 569 (199, 934)* | 0.00 (-0.04, 0.04) | Dominated | 8% | 0% |
|  | Assuming all injections were bevacizumab | 7,947 (126) | 1.57 (0.02) | 7,256 (151) | 1.57 (0.02) | 690 (378, 998)* | 0.00 (-0.04, 0.04) | Dominated | 4% | 0% |
|  | Assuming all injections were aflibercept | 16,105 (486) | 1.57 (0.02) | 17,190 (555) | 1.57 (0.02) | -1,085 (-2,276, 117) | 0.00 (-0.04, 0.04) | 976,216 SW | 94% | 96% |
| **Week 0-192** | | | | | | | | | | |
| Base case |  | 20,630 (746) | 2.88 (0.05) | 21,034 (851) | 2.88 (0.05) | -404 (-2,092, 1,282) | 0.01 (-0.10, 0.11) | Dominant | 67% | 67% |
| Discount rate | 0% | 21,456 (794) | 3.03 (0.05) | 21,885 (906) | 3.02 (0.06) | -429 (-2,213, 1,363) | 0.01 (-0.11, 0.12) | Dominant | 67% | 67% |
|  | 5% | 20,302 (727) | 2.83 (0.05) | 20,696 (829) | 2.82 (0.05) | -394 (-2,043, 1,249) | 0.01 (-0.10, 0.11) | Dominant | 67% | 67% |
| Excluding concomitant medication costs | | 20,592 (744) | 2.88 (0.05) | 20,939 (835) | 2.88 (0.05) | -348 (-2,024, 1,327) | 0.01 (-0.10, 0.11) | Dominant | 65% | 65% |
| Vision Bolt-on QALYs | | 20,630 (746) | 2.81 (0.05) | 21,034 (851) | 2.80 (0.06) | -404 (-2,092, 1,282) | 0.01 (-0.10, 0.13) | Dominant | 67% | 67% |
| Excluding participants who died by 4 years (n=387) | | 21,291 (761) | 2.99 (0.04) | 21,232 (874) | 2.93 (0.05) | 60 (-1,705, 1,829) | 0.06 (-0.04, 0.16) | 970 | 82% | 48% |
| Excluding participants who died or withdrew by 4 years (n=335) | | 21,572 (823) | 3.02 (0.03) | 21,671 (917) | 2.99 (0.05) | -100 (-2,034, 1,814) | 0.03 (-0.06, 0.11) | Dominant | 67% | 55% |
| Primary analysis population: Excluding participants who died, withdrew or were lost to follow-up by 4 years (n=328) | | 21,511 (818) | 3.01 (0.04) | 21,616 (920) | 2.99 (0.05) | -105 (-2,018, 1,785) | 0.02 (-0.06, 0.11) | Dominant | 66% | 54% |
| Excluding participants completing 2-years visit after pandemic (n=252) | | 21,385 (890) | 2.88 (0.06) | 21,632 (1,080) | 2.89 (0.06) | -247 (-2,446, 1,951) | -0.01 (-0.14, 0.11) | 18,356 SW | 49% | 59% |
| Controlling for recruiting sites | | 21,550 (1,005) | 2.91 (0.07) | 22,101 (1,017) | 2.91 (0.07) | -552 (-2,215, 999) | 0.01 (-0.10, 0.11) | Dominant | 73% | 77% |
| Monitoring visit | OCT every 3 months after year 1 | 18,947 (679) | 2.88 (0.05) | 19,375 (769) | 2.88 (0.05) | -428 (-1,967, 1,115) | 0.01 (-0.10, 0.11) | Dominant | 68% | 70% |
|  | Assuming perfect monitoring visit attendance | 22,540 (712) | 2.88 (0.05) | 23,026 (792) | 2.88 (0.05) | -486 (-2,074, 1,097) | 0.01 (-0.10, 0.11) | Dominant | 69% | 72% |
|  | OCT only when participants have injections | 18,306 (809) | 2.88 (0.05) | 19,073 (920) | 2.88 (0.05) | -767 (-2,612, 1,098) | 0.01 (-0.10, 0.11) | Dominant | 75% | 79% |
| SRT price | 80% | 20,410 (746) | 2.88 (0.05) | 21,034 (851) | 2.88 (0.05) | -623 (-2,311, 1,061) | 0.01 (-0.10, 0.11) | Dominant | 73% | 76% |
|  | 120% | 20,849 (746) | 2.88 (0.05) | 21,034 (851) | 2.88 (0.05) | -185 (-1,873, 1,502) | 0.01 (-0.10, 0.11) | Dominant | 60% | 57% |
|  | 140% | 21,069 (745) | 2.88 (0.05) | 21,034 (851) | 2.88 (0.05) | 35 (-1,653, 1,721) | 0.01 (-0.10, 0.11) | 4,860 | 53% | 47% |
|  | 160% | 21,288 (745) | 2.88 (0.05) | 21,034 (851) | 2.88 (0.05) | 254 (-1,434, 1,942) | 0.01 (-0.10, 0.11) | 35,416 | 46% | 38% |
|  | Including SRT transport costs at £207/patient | 20,836 (746) | 2.88 (0.05) | 21,034 (851) | 2.88 (0.05) | -197 (-1,885, 1,488) | 0.01 (-0.10, 0.11) | Dominant | 60% | 58% |
| Anti-VEGF injections | 20% discount on anti-VEGF | 19,763 (684) | 2.88 (0.05) | 20,193 (782) | 2.88 (0.05) | -429 (-1,994, 1,139) | 0.01 (-0.10, 0.11) | Dominant | 68% | 69% |
|  | 30% discount on anti-VEGF | 19,330 (654) | 2.88 (0.05) | 19,772 (748) | 2.88 (0.05) | -442 (-1,954, 1,066) | 0.01 (-0.10, 0.11) | Dominant | 68% | 71% |
|  | 50% discount on anti-VEGF | 18,464 (595) | 2.88 (0.05) | 18,931 (683) | 2.88 (0.05) | -467 (-1,863, 926) | 0.01 (-0.10, 0.11) | Dominant | 70% | 74% |
|  | 80% discount on anti-VEGF | 17,165 (513) | 2.88 (0.05) | 17,669 (591) | 2.88 (0.05) | -505 (-1,727, 716) | 0.01 (-0.10, 0.11) | Dominant | 71% | 79% |
|  | Assuming all injections were bevacizumab | 11,310 (284) | 2.88 (0.05) | 10,687 (337) | 2.88 (0.05) | 623 (-29, 1,272) | 0.01 (-0.10, 0.11) | 86,763 | 32% | 3% |
|  | Assuming all injections were aflibercept | 32,785 (1,531) | 2.88 (0.05) | 33,984 (1,748) | 2.88 (0.05) | -1,199 (-4,550, 2,169) | 0.01 (-0.10, 0.11) | Dominant | 76% | 75% |
|  | Assuming all injections were ranibizumab | 20,235 (718) | 2.88 (0.05) | 20,779 (821) | 2.88 (0.05) | -544 (-2,188, 1,107) | 0.01 (-0.10, 0.11) | Dominant | 71% | 73% |
| *Notes:* Results were analysed on an intention-to-treat principle with missing data imputed from multivariate imputation, conditional on participants being alive. The reference year for costs was 2021-2. Outcomes for the anti-VEGF monotherapy group are for those randomised to sham SRT.1 † The probability that treatment is cost-effective is based on a £20,000/QALY ceiling ratio. Cost-effectiveness ratios marked with ‘SW’ are in the southwest quadrant, in which treatments with high ratios are considered good value for money. Dominant indicates that the SRT is more effective and costs less than Sham. Dominated implies that the SRT is less effective and more costly than Sham. CIs were estimated using bootstrapping adjusted for treatment allocation, sex at birth, days on-trial during the COVID-19 pandemic, baseline age, baseline EQ-5D utility, and baseline study eye BCVA. Mean values in each treatment group are presented for the average participant, assumed to be women aged 78 years, who had zero days on-trial affected by the COVID pandemic with BCVA of 69 letters and EQ-5D-5L utility of 0.84. *Between-group differences based on two-sided bootstrap p-values (p < 0.05). Abbreviations: SRT, stereotactic radiotherapy. VEGF, vascular endothelial growth factor. QALY, quality-adjusted life year. CI, confidence interval. SD, standard deviation. | | | | | | | | | | |

### Table S12. Potential budget impact from SRT for high-income countries globally. Adapted from Jackson et al. (2024) Table S5 ^7^

| **Item** | **Proportion of participants** | **Number of participants in high-income countries** | **Reference** |
| --- | --- | --- | --- |
| People aged 50 years and over 2023 | N/A | 489 941 685 | [World Bank: population of high-income countries aged ≥50 in 2023 https://databank.worldbank.org/source/population-estimates-and-projections](https://databank.worldbank.org/source/population-estimates-and-projections) |
| Difference per participant treated SRT vs. sham | -£404 (-£2 092, £1 282) | N/A | Table 1 STAR study costing analysis, including cost of SRT, monitoring consultations, anti-VEGF administrations, primary care and concomitant medications, and hospital care - over four years (2021/2 prices) |
| ***Budget impact for prevalent cohort*** | | | |
| Prevalence of late-stage wet age-related macular degeneration | 1·20% | 5 879 300 | [Prevalence in England: NICE resource impact template for faricimab for wet age-related macular degeneration https://www.nice.org.uk/guidance/ta800/resources](https://www.nice.org.uk/guidance/ta800/resources) |
| Proportion of people who are eligible for anti-VEGF | 85.00% | 4 997 405 | [NICE resource impact template for faricimab for wet age-related macular degeneration https://www.nice.org.uk/guidance/ta800/resources](https://www.nice.org.uk/guidance/ta800/resources) |
| Number of prevalent people likely to have SRT | 22% | 1 099 429 | In the original INTREPID study, 272 participants were screened, of whom 230 were eligible. The STAR study was then based on the best responder subset, which comprised 26% of the INTREPID population. We therefore assumed that 22% of participants are eligible. |
| Cost saving over 4 years - prevalent participants | N/A | £444 169 316 | Cost saving per participant (£404) multiplied by number of potential participants |
| ***Budget impact for incident cohort*** | | | |
| Incidence of late-stage wet age-related macular degeneration | 0.19% | 930 889 | [Incidence in England: NICE resource impact template for faricimab for wet age-related macular degeneration https://www.nice.org.uk/guidance/ta800/resources](https://www.nice.org.uk/guidance/ta800/resources) |
| Proportion of people who are eligible for treatment | 85.00% | 791 256 | [NICE resource impact template for faricimab for wet age-related macular degeneration https://www.nice.org.uk/guidance/ta800/resources](https://www.nice.org.uk/guidance/ta800/resources) |
| Number of incident people likely to have SRT each year | 22% | 174 076 | In the original INTREPID study, 272 participants were screened, of whom 230 were eligible. The STAR study was then based on the best responder subset, which comprised 26% of the INTREPID population. We therefore assumed that 22% of participants are eligible. |
| Cost savings over 4 years after SRT-incident participants | N/A | £70 326 704 | Cost saving per participant (£404) multiplied by the number of potential participants |
| ***Annual figures: Incident and prevalent cohorts combined*** | | | |
| Cost savings per year | N/A | £128 624 005 | Number for prevalent cohort plus number for incident cohort, all divided by four |
|  | | | |

# References

1. National Institute for Health and Care Excellence. NICE health technology evaluations: the manual. 31 January 2022. <https://www.nice.org.uk/process/pmg36/resources/nice-health-technology-evaluations-the-manual-pdf-72286779244741> (accessed 7 February 2022.

2. Hernández Alava M, Pudney S, Wailoo A, Chrysanthou G. Estimating the relationship between EQ-5D-5L and EQ-5D-3L. 2020. <https://eepru.sites.sheffield.ac.uk/projects/estimating-the-relationship-between-eq-5d-5l-and-eq-5d-3l> (accessed 30 March 2022.

3. Manca A, Hawkins N, Sculpher MJ. Estimating mean QALYs in trial-based cost-effectiveness analysis: the importance of controlling for baseline utility. *Health economics* 2005; **14**(5): 487-96.

4. National Institute for Health and Care Excellence. Brolucizumab for treating wet age-related macular degeneration [ID1254] Committee Papers. 2020. <https://www.nice.org.uk/guidance/ta672/evidence/committee-papers-pdf-8964536941> (accessed 11 May 2022.

5. National Institute for Health and Care Excellence. Age-related macular degeneration NICE guideline [NG82] Appendix J: Health economics. 2018. <https://www.nice.org.uk/guidance/ng82/evidence/appendix-j-health-economics-pdf-170036251093>.

6. Chakravarthy U, Harding SP, Rogers CA, et al. A randomised controlled trial to assess the clinical effectiveness and cost-effectiveness of alternative treatments to Inhibit VEGF in Age-related choroidal Neovascularisation (IVAN). *Health Technol Assess* 2015; **19**(78): 1-298.

7. Jackson TL, Desai R, Wafa HA, et al. Stereotactic radiotherapy for neovascular age-related macular degeneration (STAR): a pivotal, randomised, double-masked, sham-controlled device trial. *Lancet* 2024.

8. Jones K, H. W. Unit Costs of Health and Social Care 2022. 2022. <https://kar.kent.ac.uk/id/eprint/100519> (accessed 22 December 2023.

9. Department of Health & Social Care. Letter setting out general ophthalmic services fees from April 2022. 2022. <https://www.gov.uk/government/publications/nhs-general-ophthalmic-service-fees-and-optical-voucher-values-from-april-2022/letter-setting-out-general-ophthalmic-services-fees-from-april-2022> (accessed 17 June 2024.

10. Joint Formulary Committee. British National Formulary. London: BMJ and Pharmaceutical Press; March 2022.

11. NHS Business Services Authority. Prescription Cost Analysis – England – 2021/22. 2022. <https://www.nhsbsa.nhs.uk/statistical-collections/prescription-cost-analysis-england/prescription-cost-analysis-england-202122#:~:text=The%20cost%20of%20prescription%20items,in%20England%20was%201.14%20billion>. (accessed 29 Feburary 2024.

12. NHS Digital. National Cost Collection: National Schedule of NHS costs - Year 2021-22 - NHS trust and NHS foundation trusts. 2023. <https://www.england.nhs.uk/costing-in-the-nhs/national-cost-collection/> (accessed 3 March 2023.

13. Hernandez Alava M, Wailoo A, Grimm S, et al. EQ-5D-5L versus EQ-5D-3L: The Impact on Cost Effectiveness in the United Kingdom. *Value Health* 2018; **21**(1): 49-56.

14. Dakin HA, Wordsworth S, Rogers CA, et al. Cost-effectiveness of ranibizumab and bevacizumab for age-related macular degeneration: 2-year findings from the IVAN randomised trial. *BMJ Open* 2014; **4**(7): e005094.

15. NHS Digital. National Cost Collection: National Schedule of NHS costs - Year 2020-21 - NHS trust and NHS foundation trusts. 2022. <https://www.england.nhs.uk/costing-in-the-nhs/national-cost-collection/> (accessed 7 October 2022.

16. Curtis L, Burns A. Unit Costs of Health and Social Care 2015. 2015 (accessed 22 December 2023.

17. Times F. NHS to Trial Artificial Intelligence App in Place of 111 Helpline. <https://www.ft.com/content/aefee3b8-d1d8-11e6-b06b-680c49b4b4c0>.

18. IVAN Study Investigators, Chakravarthy U, Harding SP, et al. Ranibizumab versus bevacizumab to treat neovascular age-related macular degeneration: one-year findings from the IVAN randomized trial. *Ophthalmology* 2012; **119**(7): 1399-411.

19. Herdman M, Gudex C, Lloyd A, et al. Development and preliminary testing of the new five-level version of EQ-5D (EQ-5D-5L). *Qual Life Res* 2011; **20**(10): 1727-36.

20. Pennington BM, Hernandez-Alava M, Hykin P, et al. Mapping From Visual Acuity to EQ-5D, EQ-5D With Vision Bolt-On, and VFQ-UI in Patients With Macular Edema in the LEAVO Trial. *Value Health* 2020; **23**(7): 928-35.

21. White IR, Royston P, Wood AM. Multiple imputation using chained equations: Issues and guidance for practice. *Stat Med* 2011; **30**(4): 377-99.

22. DB R. Multiple imputation for nonresponse in surveys. New York: Wiley; 1987.

23. Schomaker M, Heumann C. Bootstrap inference when using multiple imputation. *Stat Med* 2018; **37**(14): 2252-66.

24. National Institute for Clinical Excellence. Judging whether public health interventions offer value for money Local government briefing Reference number:LGB10. 2013. <https://www.nice.org.uk/advice/lgb10> (accessed 17 June 2024.

25. NHS. Medicines information. 2023. <https://www.nhs.uk/conditions/medicines-information/> (accessed 08 March 2024.

26. Department of Health and Social Care, The Rt Hon Sajid Javid MP. England returns to Plan A as regulations on face coverings and COVID Passes change today. 2022. <https://www.gov.uk/government/news/england-returns-to-plan-a-as-regulations-on-face-coverings-and-covid-passes-change-today> (accessed 17 June 2024.

27. Cohen J. Statistical Power Analysis for the Behavioral Sciences. 2nd Edition ed; 1988.
